# A test of several parametic statistical models for estimating success rate in the treatment of carcinoma cervix uteri.

**DOI:** 10.1038/bjc.1975.259

**Published:** 1975-11

**Authors:** R. F. Mould, J. W. Boag

## Abstract

The parametric statistical models discussed include all those which have previously been described in the literature (Boag, 1948-lognormal; Berkson and Gage, 1952-negative exponential; Haybittle, 1959-extrapolated actuarial) and the basic data used to test the models comprised some 3000 case histories of patients treated between 1945 and 1962. The histories were followed up during the period treated between 1945 and 1962. The histories were followed up during the period 1969-71 and thus provided adequate information to validate long-term survival fractions predicted using short-term follow-up data. The results with the log-normal model showed that for series of staged carcinoma cervix patients treated during a 5-year period, satisfactory estimates of long-term survival fractions could be predicted after a minimum waiting period of 3 years for stages I and II, and 2 years for stage III. The model should be used with a value assumed for the lognormal paramater S in the range S = 0.35 to S = 0.40. Although alternative models often gave adequate predictions, the lognormal proved to be the most consistent model. This model may therefore now be used with more confidence for prospective studies on carcinoma cervix series and can provide good estimates of long-term survival fractions several years earlier than would otherwise be possible.


					
Br. J. (1ancer (1975) 32, 529

A TEST OF SEVERAL PARAMETRIC STATISTICAL MODELS
FOR ESTIMATING SUCCESS RATE IN THE TREATMENT OF

CARCINOMA CERVIX UTERI

R. F. MOULD* AND J. W. BOAG

Fron Westminster Hospital*, London, SW], and the Institute of Cancer Research an(l

Royal 711arsden Hospital, Sutton, Surrey

Receivoel 24 July 1975. Accepted 5 August 1975

Summary.-The parametric statistical models discussed include all those which
have previously been described in the literature (Boag, 1948-lognormal; Berkson
and Gage, 1952-negative exponential; Haybittle, 1959-extrapolated actuarial) and
the basic data used to test the models comprised some 3000 case histories of patients
treated between 1945 and 1962. The histories were followed up during the period
1969-71 and thus provided adequate information to validate long-term survival
fractions predicted using short-term follow-up data. The results with the log-
normal model showed that for series of staged carcinoma cervix patients treated
during a 5-year period, satisfactory estimates of long-term survival fractions could
be predicted after a minimum waiting period of 3 years for stages I and II, and 2
years for stage III. The model should be used with a value assumed for the log-
normal paramater S in the range S = 0-35 to S = 0 40. Although alternative models
often gave adequate predictions, the lognormal proved to be the most consistent
model. This model may therefore now be used with more confidence for prospective
studies on carcinoma cervix series and can provide good estimates of long-term
survival fractions several years earlier than would otherwise be possible.

ALTHOUGH the 5-year survival rate or, for us and if the logical framework of a
in more general terms, the m-year survival model can be shown to be valid, the
rate, determined from an m-year follow-up  evaluation of the various parameters is
of all the surviving patients, is widely  now easy. Such models do provide a
used as a criterion of success in cancer way of bringing to bear a great deal of
therapy, it is too crude and too long  valuable past experience upon the assess-
delayed a statistic to be a satisfactory  ment of new short-term results. Indeed,
way of comparing alternative treatments they often allow a useful prediction of
during the working life of a surgeon or longer term results to be made from the
radiotherapist. Even if this rate is as-  available short-term data. Moreover, the
sessed by the actuarial (i.e. life table)  detailed classification they demand can
method, it still requires that a consider-  be of help in assessing whether an improve-
able proportion of all cases shall have  ment in m-year survival rate is due to
survived the full m-year term. Statistical long-term cures or merely to protracted
models which attempt to allow for the  survival with cancer. Confidence in any
delayed mortality during the follow-up  such model must, however, be built up
period have rarely been used, partly  by its successful use on actual follow-up
perhaps because when they were first data. This can be done retrospectively
put forward (Boag, 1949; Berkson and  by using records of cases treated many
Gage, 1952) the tedious computation in- years ago and followed up at intervals
volved had to be done by hand. The    until death with or without cancer or
digital computer has solved that problem  long-term symptom-free survival had been

:37

530                       R. F. MOULD AND J. W. BOAG

proved. However, detailed case histories  several possible statistical models which
are necessary and these are not readily   have been   suggested, and  some new
available in sufficient numbers or over  ones.

long enough periods   certainly not in a     These tests have been made in 2
single cancer centre. The Regional Can-   stages-firstly, the actual survival time
cer Registries which provide data for    distribution for each group of patients
the Office of Population Censuses and     examined has been compared, for each
Surveys   do,  indeed, have    data  in   model, with the postulated   analytical
bulk  but not in    sufficient detail for  form, choosing the model parameters to
testing a parametric model, and since    give the best fit, and assessing the good-
1970 they   are no longer required to    ness of fit achieved by a x2 test. Second-
record the disease stage (O.P.C.S., 1970). ly, accepting only the limited survival
Also, there is no uniformity of data      data which would have been available
collection, storage and retrieval within  a few years (2, 3 or 4 years) after the end
the medical records departments of dif-   of the 5-year period under review, the
ferent hospitals.   The  only  accurate   models were used to predict the 7-year,
method of obtaining the essential treat-  10-year or 15-year survival fractions as
ment and follow-up information is to     well as the    proportion  of long-term
consult the original hospital case records  cures " C ". These predicted values were
at a number of centres.                  then compared with the observed 7-, 10-,

For the present study on a single     or 15-year results, taking account of the
site  carcinoma  cervix  uteri-material  standard errors of both predicted and
has had to be gathered from     6 large  observed results. The rationale of this
cancer centres, covering a 25-year period.  " prediction " and "proof" test is illus-
We have used this material to test       trated in Fig. 1.

Time Scale. Years.

Year when initial
patient  treated

Year when final                                       Yawh      follow-ip
patient treated                                       details were

y        ediction                           transcribed from the
Y                                               patient case  notes

P (Yf + n)                (T+ m)                  Y

Treatment                                                  1969   1971

I nterval

FIG. 1. Validation of a statistical model.

ESTIMATING SUCCESS RATE IN TREATMENT OF CARCINOMA CERVIX UTERI 531

MATERIALS AND METHODS               TABLE II.-Grouping by Stage, Hospital
Patients                                               and Treatment Period

Two major factors which affect prognosis          Treatment          Total Reference
in cancer are the site of the disease and the  Hospital  period  Stage  cases  letter
stage it has reached before treatment. For    CHUM     1945-49      I    138    A
this study we have therefore selected a       CHUM     1950-54      I    179    B
single site and have separated cases into     z        1945-49      I    101    D
stage groups before analysis. Between 1969    z        1950-54      I    127    E
and 1972 some 6000 case histories were        Z        1955-59      I    292    F
examined of women treated between 1925        N        1955-59      I    553    G

and 1962 at the hospitals listed inCHUM             1945-59      I    582    AA
and 1962 at the hospitals listed in Table 1.  z        1945-59      I    520    BB

TABLE I.-Carcinoma Cervix Case His-           H        1945-49     II     68    H

C        1945-49     II    110    I

tories Available for Analysis          mu       1945-49     II     97    J

MU       1950-54     II     86    K
Treatment     C       1950-54      II   144     L
Stage         Hospital           years      H        1950-54      II   143    M
I-IV   Middlesex                1925-62     C        1955-59      II   117    N
I-IV   Royal Marsden and Chelsea  1929-62   MU       1955-59     II    123    0
I-IV   University College       1941-62     H        1955-59      II   152    P
I-IV   Hammersmith              1942-62     CHUM     1045-59      II  1040    CC
I      Christie, Manchester     1945-59

I      Oslo                     1955-59     CHUIM    1945-49    III    170    Q

CMU      1950-54    III    115    R
H        1950-54    III     90    S
For those London hospitals included, all      mu       1955-59    III     77    T
case records still available were reviewed    H        1955-59    III     78    U

C        1955-59    III     78    v

and these data are therefore complete in      CHUM     1945-59    III    608    DD
the sense that no further data exist at these

hospitals for carcinoma cervix treatments     CHUM     1945-59 I+II+III 2230    EE
before 1962. It can be assumed that data
before 1945 are fragmentary inasmuch as

many of the early records have either been  The stage IV   group was also small and
lost or destroyed. In view of this uncer-   wasgathered only fromtheLondonhospitals
tainty, only post-1945 records have been    Stage IV  is not of any value for testing
used to test the various statistical models  predictive models but we have tested its
and the post-1945 era has been subdivided   conformity with the survival time distribu-
into three 5-year treatment periods-1945-   tion of the unsuccessfully treated cases.
49, 1950-54 and 1955-59. Since the records  The letters C, H, U, M, Z and N refer to the
were examined in the period 1969-72 there   6 hospital centres of Table I.
was a minimum follow-up period of 20 years
for the 1945-49 group, of 15 years for the

1960-54 group and of 10 years for the       Methods

1955-59 group.                                 (a) Construction of a statistical model.-

The stage I groups from the 4 London     When a large group of patients is treated for
hospitals were much smaller than the stage II  cancer, a temporary remission is achieved in
or stage III groups, and therefore additional  many cases and in some there is no return of
data for stage I was obtained from   Man-   the disease before the death of the patient
chester and from Oslo for the period 1945-59.  from  some other cause many years later.
Table II shows the grouping of cases avail-  Although one cannot claim a certain " cure "
able to test the validity of the different  in any individual case, in view of the residual
statistical models. For stages I-Ill there  risk of recurrence, it is surely not unduly
are data from at least 2 different single or  optimistic to attempt to distinguish and
grouped centres for each 5-year treatment   estimate a " proportion cured " by appro-
period, except for stage III during the     priate statistical techniques applied to any
period 1945-49 where only a single group    large group   of patients. Two kinds of
from  the London hospitals was available.   model have been proposed and we shall

532                       R. F. MOULD AND J. W. BOAG

TOTAL CASES

TREATED

r

PROPORTION CURED                                 PROPORTION NOT CURED

N  (t)

Death rate
of cases
who die
with Ca.

,      /              ~~~~~~~~Cervix .

present.            Tail

olA rea/

t     SURVIVAL TIME (t)
T

N (t). dt = 1  and Q =j N (t). dt
FIG. 2. Statistical modlel, Type I.

test both kinds against the data on cervix       _ log t- M
cancer listed in earlier paragraphs.     where x -

The first kind of model explicitly recog-

nizes the existence of a proportion cured,     N (t) =x . exp (-axt)         (2)
denoted by C, and assumes that only the

complementary fraction (1/C) is at risk for    IN(t)  y   t exp    Y. ,It)   (3)
a recurrence of cancer although, of course,           12
all are at risk for other causes of death

(Fig. 2). To complete a model of this          N(t)   No . t . exp (-y . tc)  (4)
kind, it is necessary to find an appropriate
formula for the distribution of survival

times~~~~ ~ whc  ocu  wihi  thsfato    Table III lists the sources of these

several proposals and the methods of analysis
(1/C). The general shape of the curve is  used.

skew, the mortality from   persistent or    The second type of model (Fig. 3), was
recurrent cancer reaching a peak during the  first put forward by Haybittle (1959) and
first one or 2 years after treatment and

declining gradually thereafter. Several ana-  was called  by  him  the  " extrapolated
lytical forms for this curve have been pro-  carial" mode    postulateslan  anayi

pose, amng tem  he lgnoral crvecal form for the gradually declining cancer
posed, among them   the lognormal curve  mortality which affects the whole group of

(equation 1), the negative exponential (equa-  patients subsequent to treatment. Although
tion 2) and the skew exponential (equation  the " cured " group was not explicitly
3). The latter is a particular example of postulated, it is implicit in this model also,
a family of skew curves with the general  since

equation 4.                    who~~~~lne the declining mortality causes the
equation*4.                     whole group of patients to approach asymp-

N(x)   -    exp (-1 X2)       (1)  totically a fixed fraction of its original size,

/27r        2               which then survives from cancer indefinitely.

ESTIMATING SUCCESS RATE IN TREATMENT OF CARCINOMA CERVIX UTERI 533

TABLE III.-Parameters of the Various Models

Method of

Model                                                                    determination
type             Model                  Reference        Parameters     of parameters

I    Lognormal                  Boag (1949)             M (logtime) Maximum likelihood

S
C

Negative exponential       Berkson and Gage (1952), cx (time)-'  Least squares or

.HIaybittle (1959)    C            maximum likelihood
Skew exponential           Mould (1973)            y (time)-'/2 Maximum likelihood

C

II    Extrapolated actuarial     Haybittle (1959)       ,B (time)-'  Maximum likelihood

K            for K and 8

C            C = exp (-K/fl)
Haybittle (1965)       ft (time)-'  Maximum likelihood

C

Skewed extrapolated actuarial The current paper    E (time)-'  Maximum likelihood

C

Total Cases Treated      where N is the number surviving to time t

and  0(t) is any   function  satisfying  the
conditions

En~~~~~~~~~~~~~0

0 +(t) dt = 1 and +(t) -O0
as t -x oc then we can deduce that

MORTALITY CU RVEN                         N    g     . * , /4t) dt

JN, [oC]             0
that is

Death                 %                              log         log     .D 4)(t)
Rate of

all cases        8    \   ss                 where D is the integral of b. Therefore as
in the series               E                t-> oo    -* and so

N_

N0 _> C

Survival Time, t.       and C measures the     ultimate  cure rate.

Thus we may write

SURVIVAL CURVE                         NN -  (C)+@(t)
1                              where

Su rvival I                                               4)(t)=| 0(t) dt.
Fraction                                                          o

when causes                                     The function b can therefore be chosen
of death other                               with  considerable freedom  to   provide  a
than ca.cervix               _       _ _?good fit to the observed or expected dis-
are ignored                              c   tribution of survival times. Ca deaths in any

interval are then

Survival Time, t.           N1 - N2 = N0{(C)+D(ti) -(C)+O(t,)

or, we may express the same relationship
FIG. 3.-Statistical model, Type II.    by saying that the probability that an

individual patient in the treated group
If we put                                 shall die of cancer in the interval (tl, t2) is

- dN =N . log (1V0(t) dt             (N1 - N2)/No, i.e. is

acJ                            {(C)+(D(ti) -(C) (W

534                             R. F. MOULD AND J. W. BOAG

Patient follow-up group CODE:
1 = Died, ca. cervix present.

2 = Died, intercurrent disease.

3 = Alive with no sign of recurrence.
4 = Alive with ca. cervix present.

5 = Died, no data on cause of death.
6 = Died, intercurrent disease, but

no available data about ca. cervix
9 Died indirectly due to treatment.

10 - Died, second primary cancer unrelated

to ca. cervix was present.

Appa r      Disease    Survival Time        Terminal Disease
4 = Free Time    D   w    lt    n       be
Initial Planned Treatment            (0 reZ d     alive neore quaterof th.

CODE : R - Radiotherapy only,   ;       eoddt h     ers   ure-ot)         _
S - Surgery only, R + S = Radiotherapy

and Surgery.                    ,                                          -Age
Histology CODE: 1 - Squamous cell,                         1   29.

2=FeCarcinoma, no further detailss f             t                        [lilt card system.

3 = Biopsey negative, no recorded   b   tied  r     (   Data 8toraq   and mri       th       sur-
histology, 4 = Adenocarcinoma, i(u                                                wihe   dgtal
5s Transitional cell, 7 = Any other e  c                 t          t

histology not covered by codes 1 - 5  Hospital Treatment Annual dates for   le

CODEl   Site.    Start of initial planned

2a= C   CODE    treatment, estimated date

3 = U  14 a Cervix of recurrence (if any),

4 = H            Date when last known to be
6 a   Z          alive - or - Date of death.

7 - N            CODE : 1 = 1931, 2 - 1932 ..

8 s M       ~~~. . .. 12 = 1942, .... .32 1962. . .

FIG. 4.t Parameter codes for the punched card system.

Various expressions have been tried for           (b) Data storate and retrievaa .  This sur-
the  function  +(t).  Haybittle (1959, 1965)      vey was undertaken at a time when digital
chose 0(t) =b    .    exp (-agt) and called this  computers were readily      available for cal-
the " extrapolated actuarial " model. We          culation but much less available for data
shall test this model in various ways in later    storage and    retrieval  for    these  functions
paragraphs using our carcinoma cervix data.       make quite different demands on the machine.
This form    for 0(t) implies that cancer mor-    The number of cases we had to examine was
tality in the treated group will be a raximum       not too largeq some 6000    to be dealt with
at t = 0, that is, immediately after treatment,   manually on an edge-punched card system
whereas all clinical experience indicates that    and we chose this for our data base, extracting
mortality is low at t -0 and rises to a peak      all the relevant information for each patient
which occurs at anything from a few months        onto a single 8 X 5 inch card of the design
to a few   years after treatment, depending       illustrated in Fig. 4 which shows the Formica
on the site and stage of the disease.      The    template used to assist punching the data.
various skew curves tried out in the Type I       All the information for each patient was thus
models to fit the distribution     of survival    in  an  immediately    visible  form, making
times may be tested again as hypotheses           checking easy, and the cards could be sorted
for +(t). In an attempt to find a simple          quickly into their various groupings by means
single  parameter representation      for  0(t),  of the edge-punched holes and slots.       Sur-
we have tested the form 0(t) =   E 2texp (-,Et),  vival time data derived from          these card
calling, this the skewed extrapolated actu-       sorting operations were punched onto paper
arial. CDtape as required and entered into the com-

ESTIMATING SUCCESS RATE IN TREATMENT OF CARCINOMA CERVIX UTERI 535

puter in this form for the necessary statistical  other models uses only  2. This extra
estimation procedures.                    parameter makes the distribution curve more

(c) Estimation of the parameters of the  flexible and thus facilitates a good fit with
statistical models being studied.-In the first  the observations, but another consequence
type of test referred to in the introduction,  is that the standard errors of the para-
namely, testing the " goodness of fit " of a  meter values increase so that the estimate
completed histogram of survival times with  of any one parameter-such as C -is less
some postulated analytical distribution, the  stable. A  2-parameter model is clearly
best values of a single parameter of the  simpler than a 3-parameter one and it is
distribution could be estimated directly by a  shown below that the parameter S in the
standard " least squares " method.        lognormal can often be treated as a constant,

When 2 or 3 parameters have to be esti- thus converting this model also to a 2-
mated simultaneously from the incomplete  parameter one. In the present survey of
data of a treatment series-incomplete be-  ca. cervix uteri, S = 0 40 fits practically all
cause further deaths with cancer will still be  our data.

added to the histogram of survival times-    (d) Extrapolated survival fractions.-The
more general estimation methods must be   various models may be used simply as a
adopted and we have chosen the " method of  framework  for extrapolation  instead  of
maximum   likelihood " (Lea, 1945; Fisher,  attaching absolute significance to the quan-
1922).                                    tity, C, as " proportion cured ". Thus the

The logic of this method is to take as  " m-year survival fraction " may be cal-
"best " values of the parameters those    culated from the model (Fig. 2) as:
which would yield the highest chance of           S.F.(m) = C + (1 - C)Q(m)
obtaining a sample of the type actually

observed, when the calculation of probability  even when the parameter estimates are
is based on the chosen statistical model.  based on survival data for less than m
The detailed algebra involved in applying  years. This is the " prediction " indicated
maximum likelihood to the several models in  in Fig. 1. The " proof" is then the actual
Table III has been given elsewhere (Mould  survival fraction observed after m  years
1973). The iterative computations involved  follow-up when causes of death other than
in solving the equations have been carried  cancer are excluded, this fraction being
out by writing programmes either in BASIC  evaluated by the actuarial method as de-
or in FORTRAN IV for each of the models.  scribed by Greenwood (1926), Merrell and

Four mutually exclusive follow-up groups  Shulman  (1955) and Cutler and Ederer
can be seen in the top right-hand area of  (1958).
Fig. 4 with codes numbered 1, 2, 3 and 4

respectively. Groups 5 and 6 occur when                   RESULTS

follow-up data in the patients' notes are  (a) Testing  the analytical form  of the
incomplete: further supplementary informa-  survival time distribution
tion, if eventually available, may require

the transfer of a patient from these groups  Agreement between the observed sur-
to one of the Groups 1, 2, 3 or 4. If no  vival time distributions and the proposed
additional information is forthcoming, a  analytical formulae was tested by group-
decision on this transfer must be taken on  ing survival times into equal logarithmic
the basis of the last detailed follow-up report. intervals* and comparing observed with
The small Group 9 may be combined with    theoretical numbers in each interval by
Group 1 and the even smaller Group 10     means of a X-squared test for the 27
combined with Group 2, of which it is a       .       .  iT

special case. Thus we can allocate all the  hospital series in Table II.  The theor-
cases to one or other of the first 4 mutually  etical parameters were varied stepwise
exclusive follow-up groups.               in the programme until a minimum     x-

The lognormal model employs 3 inde-    squared value was found and the computer
pendent parameters, whereas each of the   then  printed  out this value together

* Basically the groups were 0-6, 6-9, 9-13-5, 13-5-20-25, 20-25-30-5, 30 5-45 5, 45-5-68-5, 68-5-
102-5, 102-5-153 -5, etc. but for small sample series these groups were sometimes combined in pairs.

536                        R. F. MOULD AND J. W. BOAG

TABLE IV.-Goodness of Fit of Data to the Skew Exponentials

P levels for different values of C
Notation: @ signifies P > 0 05

-signifies P < 0 05

No. of cancer Reference letter ,A__                       _       _

Stage    deaths     (see Table II)  ?=1 00 C=0-67 C=0-50 C=0-40 1=0-33 C=0-29 C=0-25

I       55          A           -      -      -      -      -

I       61          B                  S-
I       86          C           -                                         -
I       38          D                                -      -      -      -
I       37          E           -

I       94          F                  (D            (-                   -
I      157          G           -      -      0      -      -      -      -
1      202          AA          -      -      0      0      03            -
1      169          BB                 0      0      -

I  No. of series for which a good fit  4  6    8      5      5      5      2

to the data is obtained, P > 0 * 05

II       36          H           -      -      0      )      (0     0      -
II       63          I           -      -             (-      03    03    0D
II       62          J           -      -                    D 0    (0     0
II       50          K           -             0      0      0      0 (    0
II       85          L           -      0      03            0      0      0
II       78          M           -      (0                   (      0 3    -
II       65          N           0      0      -      -      -      -      -
II       72          0           0      0      0      0      -      -      -
II       79          P            00

II      590          CC          -      -      -      -      -      -      -
II  No. of series for which a good fit  3  6   7      8       7      7      4

to the data is obtained, P> 0 * 05

III      133          Q          -      -      -      -       -      -      -
III      96           R          -      -      -       0      0      03

III      65           S          -             0      (      (D     (D      -
III      54           T          0      0      0       -      -      -      -
III      59           U          -      0      0      0       -      -      -
III      66           V          -              0      0      0        03
III     473           DD         -       -      -      -      -      -      -
III  No. of series for which a good fit  1  4   4      4      3      3      2

to the data is obtained, P > 0 * 05

I+II+III 1265           EE          -      -      -      -      -      -      -

In each case the symbol ((D or -) in the Table gives the result for a minimum chi-squared goodness
of fit test, for the data on that horizontal level and the skew exponential dlistribution at the head of the
vertical column.

with the corresponding values of the       are tested against the data from  the 4
parameters-M and S for the lognormal,      London hospitals, Manchester and Oslo,
,8 for the negative exponential and y for  the results are those shown in Table IV.
each  member of the family      of skew   The data in this table are for patients
curves given by equation 4. We tried      treated in the 5-year periods 1945-49,
7 members of this family with C defined    1950-54, 1955-59 and followed up until
by the formula:                            1969 so that the minimum       follow-up

2/(I - r)               period was 10 years, which gives some

assurance that the tail of the distribution
where r is integral and 1 : r ; 7. This   of recurrences is adequately represented.
restriction ensured that integration  of   The C value which fits the largest propor-
equation 4 would lead to a complete       tion of the individual stage groups is
gamma function and would therefore be      C = 0 5.  C- 067 and ( = 0 40 also pro-
easily evaluated.                         vide reasonable fits but curves C       1

When the skew     exponential curves   and - = 0-25 provide poor fits to the

ESTIMATING SUCCESS RATE IN TREATMENT OF CARCINOMA CERVIX UTERI 537

STAGE I |                                    STAGE I I

C 8     A                                       6

6 -6

75                        -                  ?   3

,o    _ _  __._____________                   .5    X
>U                                                     Y

N=138

-    .-                 --                       .3

3 J
75

= f                                          c= it se

-I  ~ ~ ~ ~ ~~~~~~evx cacnoa

.)6         N   7                            I.3

L.~~~~~~~~~~~~~~~~~~~1

-.2

VI                I  I  I  I6I                                    I  I3

S= -25 30O*35 40 45 5O                       S=*. 25 30O.35 40O.45 5O

Value assumed for S                         Value assumed for S

using the Lognormal Model                    using the Lognormal Model

N=Total cases in series

Minimum follow -up period = * 4 years,X 3 years,A?2years.

FiG. 5.-Comparison of observed and predicted 10-year survival fractions for stage I and stage II

cervix carcinoma.

538                          R. F. MOULD AND J. W. BOAG

data.   We have therefore concluded from       complete London hospital series for 1945-
Table   IV   that for   carcinoma    cervix,   59 are combined, the data are not fitted by
C-0 5 is the best choice of exponent          any skew    exponential curve, nor indeed
for the skew    exponential model of the       by any lognormal or negative exponential
survival time    distribution  in  follow-up   curve either.  For other sites also, if the
Group 1 (see Fig. 4).    We have noticed       data  comprise   a  mixture    of different
that if a skew    exponential distribution     stages, it is   not usually    possible  to
is chosen, many published observational        obtain a good fit to any of these dis-
data including sites other than the cervix,   tributions.

are also best fitted by putting     C    0 5      In Table V the lognormal and negative
(Boag,   1948, 1949; Wood       and   Boag,   exponential curves are fitted to the same
1950; Smithers et al., 1952; Haybittle,       observational data, again using minimum
1959; Ronnike, 1968; Sorensen, 1958).         x2 to fix the best values of the parameters.

It is noticeable that when       all the   It is seen that the 2-parameter lognormal

TABLE V.-Goodness of Fit of Data to the Lognormal and Simple Exponential Distributions

P levels for different distributions

Notation: ? signifies P > 005

- signifies P < 005

No. of cancer  Reference letter                    Negative

Stage       deaths        (see Table II)    Lognormal      exponential

I            55             A                 -
I            61             B

I            86             C                 3

I            38             D                                -
I            :37            E
I            94             F
I           157             G

I           202             AA

I           169             BB                3-
I       No. of series for which a good fit    8               5

to the data is obtained, P > 0 * 05
II           36              H
II           63              I

II           62              J                (D
II           50              K

II           85              L                 3
II           78              M
II           65              N
II           72              0
II           79              P
II          590              CC

II       No. of series for which a good fit   10              8

to the data is obtained, P > 0 * 05

III          133              Q
III           96              R
III           65              S
III           54              T
III           59              U
III           66              V

III          473              DD

III       No. of series for which a good fit    7              2

to the data is obtained, P > 0 * 05

I+II+III      1265             EE                -              -

In each case the symbol (03 or -) in the Table gives the result for a minimum chi-squared goodness
of fit test, for the data on that horizontal level and the distribution (lognormal or simple exponential) at
the head of the vertical column.

ESTIMATING SUCCESS RATE IN TREATMENT OF CARCINOMA CERVIX UTERI 539
TABLE VI.-Summrary of the Results for the Minimum Chi-squared Goodness of Fit Tests

No. of series for which a good fit to the data is obtained, P> 0 * 05,  Total

for different distributions                 number

of series
The general                                                        tested
lognormal                          Skew exponentials               for a
with M and Negative ,                                               given
Stage  S variable  exp.  ?=1O00 C=0-67 C=0 50 C=040 C=0 33 C=0-29 C=0-25 stage

I      8         5       4     6      8      5      5      5      2      9
II     10         8       3     6      7      8      7      7      4     10
III      7         2      1      4      4      4      3      3      2      7
1+11+III    0        0       0      0      0     0      0      0      0      1
Totals     25       15       8     16     19     17     15     15     8     27

In each case the figure in the Table gives the number of series for which a good fit to the data was
obtained, P> 0 05, for the stage on that horizontal level and the distribution at the head of the vertical
column.

curve provides a good fit to all but one  dictions for long-term  survival fractions
of the 26 samples of data grouped indi-   for many carcinoma cervix series.
vidually by stage while the negative
exponential fits only 15 of them  satis-

factorily, Table VI.                     (b) Estimation of the long-termp survivors

When the lognormal is reduced to      when a 10-year mnimum follow-up interval
a single variable curve by fixing S equal  is available

to 0 40, it still provides an adequate fit   With   follow-up  data  available in
for 20 of the 27 series of data. When     1969-71 the observation periods ranged
S is fixed and equal to 0*35, the lognormal  from 10 years to 25 years and the actuarial
fits 12 series and when S is fixed and    method of calculating long-term survival
equal to 0 45, it fits 24 series. Moreover,  should, and does, converge towards an
when the model is used for prediction,    estimate of " cure rate ". We have taken
as we shall see later, the    predicted  the value at 20 years subsequent to treat-
value changes little in the range S equals  ment as this asymptotic value, with
0 30-0A40.                               which the estimates of "cure rate"

In testing the distribution of survival  based on each of the parametric models
times given by " extrapolated actuarial "  can be compared.

and similar models, one has to determine     In addition to this comparison of
first the best values of the 2 parameters  "cure rates " our computer programme
by fitting the model to the whole of the  calculated for each of the 22 groups of
data and then, using these parameter      cases in Table II, the expected survival
values, to calculate the expected number  fractions at times 5, 6, 7, 8, 9, 10 and
of cancer deaths in each interval along   15 years after treatment using both the
the time scale for comparison with the    actuarial method and each of the 5 para-
numbers observed. This we have done       metric models of Table III. A detailed
for the original Haybittle model and for  listing of all these results (except the
our modification of it but the results   skewed extrapolated actuarial) is given by
of a X2 test showed that the original    Mould (1973).

Haybittle model provided an adequate         Table VII compares "cure rate"
fit for only 12/27 series and the skewed  estimates for stages I, II and III carcinoma
extrapolated actuarial model an adequate  cervix based on each model with that
fit for only 9/27 series. Nevertheless, as  from the actuarial calculation. The value
will be seen later, both these type II   of one standard error of the actuarial
models (Table III) give adequate pre-    estimate is included in Table VII and it

540                            R. F. MOULD AND J. W. BOAG

TABLE VIL-Estimates of the Fraction Cured " C ", Based on the Available Long-term

Follow-up Information

20-year

survival    Estimate of the fraction cured, " C ", using different models
fraction   ,____

Total  Reference  calculated by    Lognormal with an     Skew           Extra- Skewed
cases    letter   the actuarial   assumed value for S    exp.    Nega-  polated  extra-

in      (see       method     A,                        with    tive    actu-  polated
Stage series  Table II)    (? 1 s.e.)  S=0-30 S=0-35 S=0-40 C=0-5       exp.    arial  actuarial

I   138       A       0-55 (0-05)   0- 57   0- 57   0-56    0- 57   0.55    0- 54   0- 57
I   179       B       0-63 (0-04)   0-62    0-61    0-60    0- 59   0-60    0-58    0-62
I  265        C       0-63 (0-04)   0-66    0-66    0-65    0-67    0-64    0-63    0-65
I   101       D       0-62 (0 05)   0-62    0-61    0-60    0-61    0-61    0-62    0-61
I   127       E       0-69 (0-04)   0-69    0-68    0-67    0-67    0-67    0-67    0-68
I  292        F       0-66 (0- 03)  0-66    0-65    0-63    0-63    0-63    0-63    0-65
I   553       G       0-68 (0-03)   0-71    0-71    0- 70   0- 71   0-69    0-69    0-71

II    68       H       0-42 (0- 07)  0- 43   0- 43   0-42    0- 43   0-41    0- 40   0-41
II   110       I       0 37 (0-05)   0- 40   0- 40   0- 39   0- 39   0-38    0-36    0- 37
II    97       J       0 33 (0-05)   0-36    0-36    0- 35   0-35    0-35    0-33    0-33
II    86       K       0- 37 (0- 06)  0- 37  0-36    0- 34   0-36    0- 37   0- 35   0-36
II   144       L       0-38 (0- 04)  0- 39   0-38    0- 37   0-38    0- 37   0-36    0-38
II   143       M       0- 34 (0- 06)  0- 43  0- 43   0-42    0-42    0-42    0- 40   0-41
II   117       N       0-41 (0- 06)  0- 44   0- 43   0-42    0- 43   0- 43   0-42    0- 43
II   123       0       0-38 (0-05)   0- 44   0- 43   0-41    0-41    0- 39   0- 37   0-42
II   152       P       0- 43 (0- 04)  0- 44  0- 43   0-41    0-41    0- 40   0-38    0- 43
III   170       Q       0-18 (0- 03)  0-20    0-20    0-20    0-20    0-20    0-21    0-17
III   115       R       0-13 (0- 03)  0-14    0-14    0-14    0-14    0-14    0-13    0-12
III    90       S       0-25 (0- 05)  0-25    0-24    0-24    0-24    0-25    0-24    0-21
III    77       T       0-28 (0- 05)  0-27    0-27    0-27    0-27    0-29    0-29    0-27
III    78       U       0-22 (0- 05)  0-20    0-20    0-19    0-20    0-22    0-22    0-19
III    78       V       0-13 (0- 04)  0-14    0-14    0-14    0-14    0-14    0-13    0-13

In each case the figure in the table for the different models gives the estimate for "C " for the data
series on that horizontal level and for the model at the head of the vertical column.

Value of  Series for which patients have been

" n "    followed up for at least " n " years
20 years      A, D, H, I, J, Q

15 years      B, E, K, L, M, R, S

10 years      C, F, G, N, O, P, T, U, V

can be seen that the " cure rate " estimates     of some 100-150 cases.      The subdivision
derived by the other methods nearly all          of the data into stage groups is highly
lie  within  one   standard    error of this     desirable in any carefully planned clinical
actuarial   estimate.    Thus,   with   long-    trial and 5 years is a reasonable period
term  follow-up available it is clear that       for a trial if clinical interest and continuity
all these statistical models will give an        of plan    are  to   be  maintained.     Any
acceptable estimate of C.       The 3 para-      suggested modifications in treatment tech-
meter lognormal model requires for sta-          nique can then be applied without too
bility a larger number of cases than are         long  a delay.    Standard   errors of this
available in these separate quinquennial         magnitude must therefore be regarded as
groups, but the     2-parameter lognormal        typical in most stratified clinical trials.
is satisfactory for any fixed value of S         To reduce the error by a factor of /2
between 0-25 and 0-50 (only values for           would involve doubling the sample size
0-3-0-4   are   quoted    in  Tables).   The     and  in  this survey    we have     reviewed
standard   errors   in  " C "  were usually      some   2000  case   histories of carcinoma
close to   0-05 for the values of C       en-    cervix from   the 4 London centres alone.
countered    and  the   small sample     sizes   Clinical trials in cancer therapy are very

ESTIMATING SUCCESS RATE IN TREATMENT OF CARCINOMA CERVIX UTERI 541

TABLE VIII.-Estimates of Stage I and Stage II 10-year Survival Fractions and Stage III

7-year Survival Fractions, Based on the Available Long-term Follow-up Information

10-year            Estimate of the 10-year survival fraction,
survival                    using different models

fraction                             A-

Total Reference  calculated by   Lognormal with an     Skew          Extra- Skewed
cases    letter  the actuarial   assumed value for S   exp.   Nega- polated  extra-

in      (see      method          --                 with     tive   actu-  polated
Stage series  Table II)   (? 1 s.e.)  S=0 30 S=0 35 S=0 40 4=0 5     exp.    arial actuarial

I   138      A       0-63 (0 04)  0-58    0 59    0-60    0-64   0-61    0-61    0-61
I  179       B       0-67 (0 04)  0-65    0-66    0-66    0-67   0-69    0-69    0-66
I  265       C       0-69 (0 03)  0-67    0-67    0-67   0-67    0-68    0-68    0-66
I  101       D       0-62 (0 05)  0-63    0-63    0-64   0-63    0-64    0-65    0-62
I  127       E       0-72 (0 04)  0 70    0-71    0-71   0-72    0 73    0 73    0- 71
I  292       F       0-68 (0 03)  0-68    0-67    0-68   0-68    0-69    0-69    0-67
I  553       G       0 74 (0 02)  0-72    0-72    0-72    0-72   0 73    0 73    0-72
II   68       H       0 47 (0 06)  0 44    0 43    0 43    0 43   0 43    0 43    0-41
II   110      I       0 44 (0 05)  0-41    0-41    0-42    0-42   0 43    0-43    0-40
II   97       J       0 40 (0 05)   0 37   0 37    0-38    0-38   0 40    0 40    0-36
II   86       K       0 43 (0 06)  0-38    0 39    0 39    0 40   0 43    0 43    0-39
II   144      L       0- 41 (0- 04)  0- 39  0 39   0 40    0 40   0 40    0- 41   0- 38
II   143      M       0-46 (0 04)  0 44    0 44    0 45    0 45   0 45    0-46    0 43
II   117      N       0 45 (0 05)  0 44    0 44    0 44    0 44   0 44    0 44    0 43
II   123      0       0-42 (0.05)  0 45    0 44    0 44    0 44   0-41    0-42    0 43
II   152      P       0 43 (0-04)  0-45    0-44    0-44    0.44   0 44    0 45    0 43

7-year survival        Estimate of the 7-year survival fraction,

fraction                    using different models

III   170      Q       0-24 (0 03)  0-20    0-21    0-22   0-22    0-22    0-23    0- 18
III   115      R       0-18 (0 04)  0-15    0-15    0-16   0-16    0-17    0-18    0-13
III   90       S       0 30 (0 05)  0-25    0-26    0-27   0-27    0-28    0-28    0-23
III   77       T       0-28 (0 05)  0-28    0-28    0-28   0-28    0-26    0 30    0-27
III    78      U       0-22 (0 05)  0-21    0-21    0-21   0-21    0-23    0-24    0-20
III    78      V       0-16 (0 04)  0-15    0-15    0-15    0-15   0-15    0-16    0-13

In each case the figure in the table for the different models gives the estimate of the 10-year (or 7-year)
survival fraction for the data series on that horizontal level and for the model at the head of the vertical
column.

Value of  Series for which patients have been

" n "   followed up for at least " n " years
20 years     A, D, H, I, J, Q

15 years     B, E, K, L, M, R, S

10 years     C, F, G, N, O, P, T, U, V

seldom  as comprehensive as that and it        actuarial and   parametric    estimates to
is evident that small treatment differences    within one standard error of the actuarial
of the order of 5% will rarely be found to     estimate.  The skewed extrapolated actu-
be significant.                                arial model gives consistently lower esti-

Using a similar format to Table VII,       mates   for the    survival fraction   than
a comparison     of the  observed   10-year   those given by the other models.        The
survival fractions with those calculated       low  values given by the skewed extra-
from   the  parametric   models for stage      polated actuarial model for stages II and
groups I and II, and of the 7-year survival    III are due to the fact that this distribution
fraction for stage group III, is given in      has  a  very   broad   peak.   The   7-year
Table VIII.     For the lognormal, skew        survival fraction   was    chosen   as  the
exponential (    = 0.5), negative exponen-     criterion for stage III as almost all cancer
tial and extrapolated    actuarial models,     deaths among patients first seen in this
there is nearly always agreement between       stage will have occurred before 10 years

542                     R. F. MOULD AND J. W. BOAG

have elapsed, so that the 10-year survival The format of Tables IX-XI is similar
fraction is virtually identical with the  to that of Tables VII and VIII.

estimate of C. Close agreement was        Tables VII and VIII give the results
observed between the extrapolated actu-  calculated from the long-term follow-up
arial and negative exponential. These  data and a single column of figures
2 models, and the skew    exponential appears beneath the heading for each
model, gave predictions for 10-year and  model. Tables IX-XI give results based
7-year survival fractions which agreed  on short-term follow-up information and
fairly well with those given by the log- the date at which the predictions were
normal model, taking a fixed value of made is defined as " n years after the
S in the range 0 30-0 45.              series closed " (see notation in Fig. 1).

Hence for Tables IX and X (for carcinoma
cervix stages I and II) there are 2 columns
(c) Eotimation of the long-term  survival Of figures beneath the heading for each
fraction when only relatively short-term  model. They correspond to predictions
follow-up data are available           made at 4 years or 3 years after the series

The data already presented confirm  closed (n = 4 and n = 3). In Table XI
that several of the statistical models for stage III carcinoma cervix, the pre-
examined can provide an accurate repre-  dictions were made at 2 years or 1 year
sentation of the life experience of car-  after the series closed (n = 2 and n = 1).

cinoma cervix patient groups when long-   Figures 5 and 6 show the results for
term follow-up data are used to estimate  treatment series A, B and C which are
the parameters of the model. It is     quoted in Table IX, and in addition
therefore of great interest to determine  results for the lognormal model with
with what accuracy the subsequent life fixed values of S ranging from 0-25 to
experience can be predicted when only  0 50 and also for the same analysis
shorter term  follow-up data are used, carried out for n  2 years. Series A,
as would normally be the case in a planned  B and C represent the combined data
clinical trial some 5-8 years from  its for stage I of the 4 London teaching
commencement. To do this, the para-    hospitals for the three 5-year treatment
meters of the statistical model to be  periods 1945-49, 1950-54 and 1955-59.
tested were first estimated by the method  A similar combination of data for stage II
of maximum likelihood from the incom-  has been annotated W, X and Y, see
plete follow-up data which would have  Table XII.
been available in our series after only a

limited follow-up period and these esti-            DISCUSSION
mated parameters were used to calculate  TiscUlm          ION
the expected 10-, 15- or 20-year survival Type I 8tatistical models

fractions. These extrapolated survival    The lognormal model.-The lognormal
fractions were then compared with the  model with 3 floating parameters, M, S
actual survival fraction calculated by  and C, requires for its stability a larger
the actuarial method from the long-term  number of cases than are available in
follow-up data on the same group of most of our quinquennial stage groups
cases (see Fig. 1). The results for the  even when long-term follow-up is avail-
several models, both type I and type II  able. This was evident in the study of
(Table III), are set out in Tables IX, X  " information content " in the original
and XI, for disease stages I, II and III  publication (Boag, 1949) and has been
respectively. For stages I and II, the  confirmed in other practical examples
10-year and 15-year survival fractions  (Wood and Boag, 1950; Smithers et
are shown but for stage III the 7-year and  al., 1952; Mould, 1973). However, the
10-year fractions were calculated instead. lognormal model with M and C floating

ESTIMATING SUCCESS RATE IN TREATMENT OF CARCINOMA CERVIX UTERI 543

es          _    q *4 coso  0 cs  t o  o
>   tdw   11OsOsOWN           OOsOstO .~~~~~~~w  >t

,   Oo;  ;ooooooo      ooooooo       O  O     t~~~~~~~~~~~~~~~~~~~~~~~~~~lz

?;   > ;: *                          t   = t   t~~~~~~~~~~~~~b

_a ~ ~~~         Q ;I

O   C  I 1 so sO ss t  s es OW OF   S      r

X~~~~~~~~~~~~~C 10 w C w wio   w r-  co to co Cw oo o  -  e=

O2   <z)   X  e  0 ||  s  s  b  s  b  s  b   t   O 0 0  O  X 0  m x  x

4~~~~~~~~~~             ~ -4a  aq  0 0000  00 0o ? 00  s)  - a3

e  ;       *  * *       O               11 0E t  *t ;~~~~~~~~~~~~~~~~~l~  t

t~~~~~~~~~~~~~~~~~o  lo  co  -4    m   0  z Q   C  XOs-

;~~~~~~~~~~~~~~~~~~~~~~~~~~~~~. O- " =1 '.4s  =  M< s  0=  e  M

Sq  a  ; * R O O O O O O O  t  O O O O O O O ~~~~~~~~~~~~~~~~~~~~~~~~~0 c._

X  *t  wX   *  *   >                e;   O to 8~~~~~~~~0 C

s O  t  X s o t Ct Q Q   Q  _ t z _ s _ X  P ;?  o  d  o~~~~~~~~~~~~~~4-

R to   1

t  m       v ROOOOOO         ^   ??????      WRR     4-D

Q~~~~~~~~~~~~~~~~~~~~~~~~~~~~~~~~~~~~~~~~~~~~~C O  Ca ;=

e~~~~~~~~~~~~~~~~~~~0   m   lo  U-X m  O4P4 qC

rC 8  o   l ce Ci eo b O e s Ci  X ~~~~~~ ce u: rs _ X o  e =  e IC  O  e

<1te m?  1OOOcF        ,OcWOn          *; tt

t3                         cy  c  4~~~~~~~  O R OOOOOOO OOOOOOO >> 0 .= U .;1~~~~~~~~~~~~~~~~4-

*s~ ~~~ e  ltmoreeme       z

u  @  t  d3  M2  1I cO eD eD Q Q cC\ b   W  eD u: s cO cO <, .  3 fv  tR  ;  t0~~~C
;3 > Q  > i R O O  O O O O ?  O O O  O O O  ,t  e  R  6

O2~~~~~~~~~~~                              ~~~ 0  Ca)  0 ;)  P. 0 * O

e 11~~~~~~~~~~~~~~~~~~0 aq P0 -X ttX Xm   Xsoes  =e m 4Ca Oe

X~~~~~~~~~~~~~~~~~~~~~~~~~~~~~~~~~~t  xo r- cn 11OcO  ' sct  v;Xeq

> t  e  O < R O O O O O O O  X  ? ? ? ? ? ?IF?I? Is ?  >-  .  -

u  u   _   fCO  urOseocur   40seset  ;  o  e :=;~~~~~~~~~~~~~~~~~~~~~~t-4 ;.  b c

4 QR:|1 D.cDsbc Cr : osc

_   EL  11 1 t  t 4 s < O oo t   t X s < c5> b b  t  P  e;  R  ?;~~~~~~~~~~~~~~~~0  e 04  0

9Df           t x o to 0 00     m             lhf-

x  e e ge et O 0000000  e e g ooooooo  _ X  <- > X e~~~~~~~~~~~=  k

tt   G) .- *,  : ,sm  O  O OO OO  >.  *,,O O  O OO O  3 u>  ~>*  :

cy                  o  B  a3  ri  a3  O  X  b O cs m a) t 14 fq e M) X Co > s s O = U): Ce0  0-
t~~~~~~ m          ct 0z 0  Cczc  J*

. c?   e=   O OO O OO O   ?? ? ?  ? ?  ~  ;  ;? O

*      ~~            ~      ~             4;) 4;)  =  >  R  I)~~~~~~~~~~~~C-  I

w~~~~~~~~~~~~~~~~~000 k8 7~    O"o>;o             . o -4.;?

0,4 ~   ~~~~~~~~~          (D >:_ Ca  > ,ok -
<6~~~~~~~~~~~~~~~~: Ca             * ?-  m  a3 m m aq  O

0. O     >
~~~~~~~~~~~~~O

Z~~~                        ~~~~~~~~~~~~~~~~~~~~~~~~~~~ 0; e *

38

544                                            R. F. MOULD AND J. W. BOAG

Id

0 -.0  I m  41? 10 aq 1-4  la I-* 00 t-           lt? to N     10 1-t  00 10

+5 Ca                                                                            > 43

0

00 t-                                     t-                  t-
-4Z

4-?
4z,

0                               0 0                           0, C' >

m              ?4
1-4                                  k >

m oo m W m r-                                  cq,..4 to m t- (D

lz

Ca

-4 -4 00 t- 10         to

? ?a " " "             "

bo
bD w

00                         ao m lo m t-        aq

40
C'D 0,

0                                   P4       k

4-D

4ZI                                                                                aq

0 >

tq

&4

10 m o      aq o lo '04

,O

4-?
co

P.,

w oo xo                                        W     aq aq             0

m     ?t I"

14)                                                       4-i     .    .   .   .   .   .   .   .   .   . 4Z

ZZ1%  -4-5                                                 0     00000000CD                     . .-        -4.'.)

0

oo lo to      ag                                           00                Z

E     " t- ?o '"? 00 ?o aq 00          Ca

It " ',?t "      " m   *              1-t " " 1-t m                   +?    X    0

P-4                   .    .   .                     -4Z     .    .   .   .   .

0 0                          T$          Ca

03

Cs
co "t aq aq             t- 00 W           m            4
ea

F-4     -4  -4---)

0 0      k.Cd

INQ                                                                                                     0

4--?                                                                                        C4Zl  $?-l

00 t- -4 00   -4 m                        t-   W        aq                  -4-)

;.4                                                                                   ;.4        C.Jz4
0                                                                                     t"   .- 19
?44                                                                                               1-4    k 4-)

00 0 t- m m t-      m                  00      aq aq r- It clt m       ce

O O O O o 0 C,:)

Ca
to         to 10                   to ull?              "it      ;.4

Ca                0 C> 0 0 0 0 (Z 0 0                         0 C C 0 CD 0 C                         >

Ca >                                              Ca > O    . . . . . . . . .                        > -4

W.,.-                6 6 6 6 <? 6 6 6 6           o.- ..                                 Ca         Ca >

14-e              -4Z .4..)                                       -4Q

>     Ca O                                      ?? > 0

Ca -  Ca                        40 N M          ;.          - t- 00       00 M        -4

CO                         -H                                                                              4Q

m                                              P4 0 Ca

-O    0

4Q

;-4

It 4.- .-,a

0 P.,                      ?4 4 P? XC) 44

1.2                                                                                               ce

P4

4Q,

4-4         -4

MoEogo.,?

0  00 (D r- W 't m t- m all               W C t-         m t- m aq                       0

0-    = -4 = X "     -* -.4 N U?             W   .-I =   ",tv - aq

Ca         Ca

-4Q Cd

ESTIMATING SUCCESS RATE IN TREATMENT OF CARCINOMA CERVIX UTERi 545

m 0

P-4 .dq M  t- r- 00 m                   m  t- I- 00

-4 aq N    q  I N                     aq 1.4    aq

PCs
z4

0 t-

?l olam         m                      C9

4-1
00    t- t- to                     t- to     m     T?

P--4  aq               -1    aq

C3

ococoo
0

P4 0

Ca       aq  00 to t-                           m  m
k         11 "? aq aq    aq m                   cl N

CD                    c

--4                                              00 00

"-I aq aq "-4   N     0   0

>

4?-

to                                           00 t-       N   m     ;.4
o  0     aq  C) 00    00 m  m         -4     P-4

-4 00 -4 00 t-      in                     00

P-4 "q aq

>

'o

04 oo     m   oo             -4     to 10 m   00 00        0  ad

II  -I   aq                  >

00
11.2                                                                                      N

N      Cd -4-D

P?          P?                                         >
pl?        0                                              1-4     ooooo<?               o

4--)

0                                                            00 00    ;4

o      11   aq        m  00    m                                          0

'..,  x       I I      ?4 "? 71 ?,                  aq aq       aq
ce                                       0

Z2               >                                                                     :2,

k

?J-Q                                                                                    0

4-')

0     CD                                       Ca                           4
$?-4                               00 t-                 m   t- t- 00 w      m
P-4                                            -4Q     --q   aq

0     C> C>

ag                                      t-    00 0  0

eq aq cq --4 aq m

PO                                                                     0  &4

-4

00 t-                           00       00 0

01       aq               -4 aq aq    cq aq

aq aq                              cq

-4           0  Ca

PO >

?4

k >
'o

Ca 0                .  .  .  .  .   .     ce    0   .  .  .   .  .  .

oooooo                   -4 -A 0 0 0 C> C 0

>     Ca                           -

>      aq 00    w  aq 00     0 0

-4 "   o  oo     aq 00              aq eq        aq aq

m L?4 0        -H eq m          cq aq               .  .   .  .  .  .    -4--D

Ca Po        666666                                                 0 0

E-1

Po
F,

0

00 00 to

ao oc                            - r- t-

t-    -4 t- t- t-                      -4 t               Ca 0

cn                                                        ce

bo ?-q

546                         R. F. MOULD AND J. W. BOAG

[STAGE I                                       [STAGE IIl
* 8                                               6 *6

.    A                                       M        W

A~~~~~~

*7 -                     ~                       .5
.2        B                                       _g

6                                                  4        N 275]i     =

5~~~S *A 25                                   3 J0*3  0*4  0S  5*3  5*4   5*5

L.                                                 .

Value assumed for S                             Value assumed for S

using the Lognormal Model                       using the Lognormal Model

N=Total cases in series

Mlinimum follow-up period = *4 years,x 3years,2years.

FIG. 6.-Comparison of observed and predicted 15-year survival fractions for stage I and stage II

cervix carcinoma.

TABLE XJJ.-Groupings by Stage, Hospital     but with S fixed at an appropriate va.lue

and Treatment Period for Fig. 5 and 6     has been shown to give an excellent fit

with survival time distributions both in
Treatment        Total Reference  the  presentheerienorm

Hospital   period  Stage  cases  letter               t series  (Table  V)landnin

CHUM      1945-49     I   138     A       numerous other series.     To show    how
CHUM       m 1950-54  I   179     B       predictions  vary     with the value of S
CHUM      1955-59    I    265     C       chosen, we have carried out predictive
CHUM      1945-49   II    2375            calculations for the 6 values from S     S25
CHUM      1950-54   II   .371     X

CHUM      1955-59   II    400     Y       to  S =O05O    in  steps of O-O5. When

ESTIMATING SUCCESS RATE IN TREATMENT OF CARCINOMA CERVIX UTERI 547

long-term follow-up data are used, the  the actuarial value. This is due to the
predicted 10-year survival fractions for  fact that, in this series, 7 patients died
the various values of S in this range do  from carcinoma cervix 12-20 years subse-
not usually differ by more than 0 03.   quent to treatment and this frequency
With short-term follow-up data, however,  of later recurrences is unusual.

extending over only 3 or 4 years subse-    For stage II carcinoma cervix, 10-year
quent to treatment, the long-term extra-  and 15-year survival fractions, there is
polated survival fractions depend more  good agreement between actuarial cal-
strongly on the value of S adopted and  culation and lognormal prediction for
in Tables VII-XI we have listed only    S   0 30, 0 35 and 0 40, and for n = 4
the estimates based on the three central years and  n   3 years. The largest
values S = 0 30, 0 35, 0 40. Figures 5  discrepancies occur when S - 0 40 and
and 6 show the trends over the wider    n = 3 years (Table X). Results for
range of S.                             stages I and II carcinoma cervix have not

For stage I carcinoma cervix, 10-year  been included for the shortest follow-up
survival fraction, there is good agreement  (n  2 years) since good agreement could
between actuarial calculation (" proof ", not be expected after only 2 years in
see Fig. 1) and lognormal prediction   these early stages where recurrence tends
(" prediction ", see Fig. 1) for fixed values  to be longer delayed.

of S equal to 0 30, 0-35 or 0 40, and for  For stage III carcinoma cervix, 7-year
both n = 4 years and n = 3 years short-  and 10-year survival fractions, there is
term follow-up information (Table IX). reasonable agreement between actuarial
The largest discrepancy occurs for series  calculation and lognormal prediction for
F, when n = 3 years and S _ 0 40. For   n = 2 years (Table XI).

this series (Table IX) no results were     A summary of these conclusions is
obtained  using the skew   exponential  shown in Table XIII. The choice of S
model since the iterative procedure did  equal to 0 30 is not recommended because
not converge, while the standard errors  when testing the analytical form of the
of the parameters in all the other models  survival time distribution of patients
were very large indeed. Evidently this  known to have died with carcinoma
series had a somewhat abnormal time     cervix present, this particular value of S
distribution.                           in the lognormal curve did not provide

There is also a good general agreement  an adequate fit to most of the data
between actuarial calculation and log-  under review  (see Results, (a)). The
normal prediction of the 15-year survival lognormal curve with S = 0 40 provided
fractions for stage I carcinoma cervix. a fit to more data than the S = 0 35
Discrepancies occur again for series F,  curve, but the data of Tables IX-XI
and also for series B with S - 0 40 andc indicate that either value is suitable for
n = 4 years (but not for n   3 years!). the purpose of predicting long-term sur-
For series A, the predicted    15-year  vival fractions.

survival fractions are always higher than  The skew exponential model.-Although
TABLE XIII.-Summary of Conditions for the Use of the Lognormal Model to Predict

Long-term Survival Fractions for Carcinoma Cervix

Minimum waiting period after a

Values which may be  5-year treatment series closes before

Carcinoma  assumed for the lognormal  use of the lognormal model  No. of cases in the
cervix stage     parameter S               (n years)           series tested*

I         S=0-35-S=0-40                n=3                  101-553
II        S =0 35-S=0 40                n=3                  68-152
III        S=0-35-S==040                 n=2                  77-170
* See Table II.

548                     R. F. MOULD AND J. W. BOAG

a maximum likelihood solution was always  estimates are of little practical value and
found for the skew exponential model this model cannot be regarded as suitable
for stage 1 carcinoma cervix when long- for stage I series with sample sizes
term follow-up data were used, the equa-  similar to those available for this study.

tions did not always yield a solution     For stage II carcinoma cervix, there
when only short-term data were available. is better agreement when n = 4 years
This failure of the iterative procedure to  than when n = 3 years, and for n = 3
converge in 3 of 7 series when n - 4   years the model is satisfactory for only
and n = 3 years, indicates that this   some half of the series studied (Table X).

model is unsuitable for predictive esti-  For stage III carcinoma cervix, the
mates on stage I series. It is perhaps  negative exponential model is unsatis-
surprising that in those cases where a  factory when n   1 year, but when
solution did exist good agreement was  n - 2 years the results are comparable
found between observation and prediction  with those obtained using the other type I
(Table IX).                            models (Table XI).

For stage II carcinoma cervix, the

results using the skew exponential model Type II statistical models

were inferior to those obtained with the  The extrapolated actuarial model.-The
lognormal model. This is particularly  extrapolated actuarial model was intro-
noticeable for short-term follow-up when  duced by Haybittle (1959) mainly for
n = 3 years. Of the 9 stage II series  carcinoma breast data but has also been
in Table X, only series P showed a large  used by him for 2 series of carcinoma
proportion of the cancer deaths occurring  cervix patients obtained from follow-up
before the analysis time n = 4 years. information reported by Sorensen (1958)
Also, most of the remaining patients   and by University College Hospital (1958).
who would eventually die with cancer   However, only estimates of C were de-
present were then already showing a    rived and the efficiency of the model for
recurrence. (This may reflect some dif-  predicting 10-year and 15-year survival
ferences in staging.) This high propor- fractions from short-term  data was not
tion of early cancer deaths has a more  discussed (Haybittle, 1960).

marked influence on the skew exponential  For stage I carcinoma cervix, it is
model than on the other models, since  seen from Table IX that the predicted
the area under the " tail " of the skew  values of the 10-year survival fractions
exponential curve is larger than that of using the type I negative exponential
the similar curves in the other models. model and the type II extrapolated
This explains the low survival fractions  actuarial model are very similar. How-
predicted for series P using this model.  ever, each of the model parameters a

For stage III carcinoma cervix, the  and , is often subject to a standard
skew exponential model is unsatisfactory  error of some 50% of its value, so these
for n - 1 year, but for n = 2 years the  models are unsuitable for use with car-
results are comparable with those obtained  cinoma cervix stage I series.

using the other type I statistical models  For stage II carcinoma cervix series,
(Table XI).                            the extrapolated actuarial model does

The negative exponential model.-For  not always give good agreement with
stage I carcinoma cervix, short-term   actuarial estimates of long-term survival
follow-up when n = 3 years, the standard  rates (Table X).

error in the negative exponential para-   For stage III carcinoma cervix, the
meter ac, was greater than 0-5a in 3 of model is unsatisfactory for n = 1 year,
the 7 series (Table IX). Thus although  but for n = 2 years the results are
there is generally good agreement between  comparable with those obtained using
actuarial calculation and prediction, the  type I statistical models (Table XI).

ESTIMATING SUCCESS RATE IN TREATMENT OF CARCINOMA CERVIX UTERI 549

The   skewed  extrapolated  actuarial term  results can be made, within cal-
model. Only one type II statistical model culable error limits.

has previously been suggested, namely,    Three parametric statistical models
the extrapolated actuarial model, and this  have previously been described, the log-
model postulated an exponential mortality  normal (Boag, 1949), the negative expo-
curve with maximum at time zero. A     nential (Berkson and Gage, 1952) and
skew curve rising to a peak within the  the extrapolated  actuarial (Haybittle,
first year or two might be expected to  1959). Each of these models makes a
represent the mortality curve with greater  different assumption about the analytical
accuracy and the skewed extrapolated   form  of the distribution of survival
actuarial model was devised as a possible times of the unsuccessful cases.

improvement. The form of this curve is   In the present study, the validity of

M(t) - (log C62t e-4          these several survival time distributions

has been assessed, using the x2 test, with
but the peak proved to be too broad and  reference to 27 different series of carcinoma
generally too far from  the origin to  of the cervix patients, drawn from several
provide a good fit for the survival time  hospitals. The patients had all been at
distribution (Results, (a)) and its use  risk for at least 10 years, having been
in a predictive model is therefore some-  treated during the period 1945-59 and
what artificial. For carcinoma cervix  followed up until 1969-71. Two further
stages I and II the predicted values were  survival time distributions were intro-
found to be inferior to those derived  duced and tested-the skew exponential
from the ordinary extrapolated actuarial and the skewed extrapolated actuarial.
model, and for stage III they were similar  A summary of the results of the tests
to those of the other models tests (Tables  for goodness of fit is given in Table VI.
IX, X and XI).                         The lognormal and the skew exponential

No doubt single-parameter skew curves  with  C- 05 give the best fit to the
could be found, possibly from the family  observed data.

given by Equation 4, which would provide  Previous tests of these parametric
a better fit but since the lognormal,  models have generally been limited to
with S fixed, has now been shown to    checking the goodness of fit of the survival
be of rather wide application (see log-  time distribution with the proposed for-
normal model, Discussion) there are little  mula, but the extrapolated  actuarial
incentive to seek alternatives which are  model has also been tested by comparing
likely to be analytically much less con-  predicted long-term survival rates with
venient.                               the observed values for carcinoma of the

breast (Haybittle, 1965).

In the present study, all 5 models
CONCLUSIONS               referred to above have been tested as
Parametric models seem to provide   predictive models for carcinoma of the
a useful alternative to the actuarial  cervix with the results shown in Fig. 1,
method of calculating survival percentages  5 and 6 and Tables IX, X and XI. When
even when follow-up data are sufficiently  all these models are tested on stage I
extensive to allow the latter method to  cases, the lognormal is consistently the
be used (Mould, 1976). They certainly  most accurate in its prediction of longer
extract more information from the clinical term results; the other 4 models sometimes
data than the crude m   year survival fail to give any satisfactory solution.
figures which are still the common form  For stage II cases, the lognormal is still
of reporting treatment results in clinical the best model but the disparity between
journals. They offer the unique ad-    this and the other models is not so
vantage that an early prediction of longer  marked. For stage III cases, where the

550                 R. F. MOULD AND J. W. BOAG

number of long-term survivors is inevit-
ably comparatively small, there are under-
standably no great differences between
the predictions from the several models.

In summary, the lognormal model,
with S fixed at an appropriate value
(Table XIII), has been shown to be of
wider validity than any of the other
models tested and to give reliable extra-
polated estimates of long-term survival
rate for the separate stage groups in
carcinoma of the cervix.

We are indebted to the following
consultant radiotherapists and surgeons
for access to the data on which this
work is based and for permission to
publish it: Mr J. B. Blaikley, Dr V. M.
Dalley, Professor E. C. Easson, Dr E. W.
Emery, Professor P. Kolstad, Dr M.
Lederman, Dr R. Morrison, Dr M. D.
Snelling and Dr L. H. Walter. We
should also like to thank Miss V. S.
Waters for secretarial assistance.

REFERENCES

BERKSON, J. & GAGE, R. P. (1952) Survival Curve

for Cancer Patients following Treatment. J. Am.
stat. Soc., 47, 501.

BOAG, J. W. (1948) The Presentation and Analysis

of the Results of Radiotherapy. Br. J. Radiol.,
21, Pt I, 128, Pt II, 189.

BOAG, J. W. (1949) Maximum Likelihood Estimates

of the Proportion of Patients Cured by Cancer
Therapy. J. R. statist. Soc., (Series B), 11, 15.

CUTLER, S. J. & EDERER, F. (1958) Maximum

Utilisation of the Life Table Method in Analysing
Survival. J. chron. Dis., 8, 699.

FISHER, R. A. (1922) On the Mathematical Founda-

tions of Theoretical Statistics. Phil. Trans. R.
Soc. A, 222, 309.

GREENWOOD, M. (1926) A Report on the Natural

Duration of Cancer. Rep. Pub. Hlth med. Subj.,
bond., No. 33.

HAYBITTLE, J. L. (1959) The Estimation of the

Proportion of Patients Cured after Treatment
for Cancer of the Breast. Br. J. Radiol., 32,
725.

HAYBITTLE, J. L. (1960) The Early EstimatioiL

of the Results of Treatment for Cancer. Br.
J. Radiol., 33, 502.

HAYBITTLE, J. L. (1965) A Two-parameter Model

for the Survival Curve of Treated Cancer Patients.
J. Am. statist. Ass., 60, 16.

LEA, D. E. (1945) The Biological Assay of Car-

cinogens. Cancer Res., 5, 633.

MERRELL, M. & SHULMAN, L. E. (1955) Deter-

mination of Prognosis in Chronic Disease Illus-
trated by Systemic Lupus Erythematosus. J.
chron. Dis. 1 12.

MOULD R. F. (1973) Statistical Models for Studying

Long Term Survival Results following Treatment
for Carcinoma of the Cervix. Ph.D. Thesis
University of London.

MOULD R. F. (1976) Calculation of Survival Rates

by the Life Table and Other Methods. Clin.
Radiol. In the press.

O.P.C.S. (1970) Report of the Advisory Committee

on Cancer Registration p. 10.

RONNIKE F. (1968) Carcinoma of the Vulva. Cure

after Operative Therapy Evaluated in Accordance
with  Boag's Statistical Method. Dan. med.
Bull. 15, 296.

SMITHERS D. W. RIGBY-JONES P. GALTON,

D. A. G. & PAYNE P. M. (1952) Cancer of the
Breast. Br. J. Radiol. Suppl. 4.

SORENSEN B. (1958) Late Results of Radium

Therapy in Cervical Carcinoma. (A Clinical-
statistical Study on 798 Patients Treated at
the Radium Centre Copenhagen during the
period 1922-29.) Acta Radiol. Suppl. 169.

UNIVERSITY COLLEGE HOSPITAL (1958) Malignant

Disease at University College Hospital 1946-50.
Shrewsbury: Wilding and Son Ltd.

WOOD C. A. P. & BOAG J. W. (1950) Researches

on the Radiotherapy of Oral Cancer. MRC
Special Report Series No. 267.

				


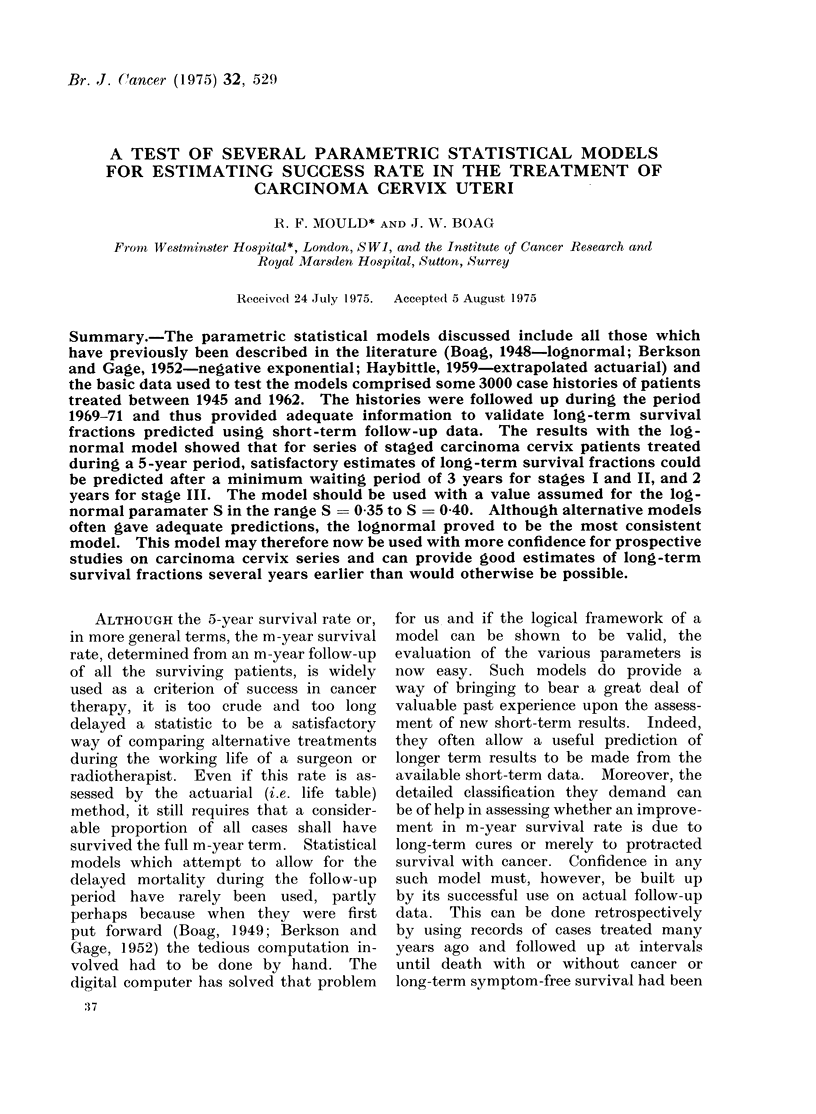

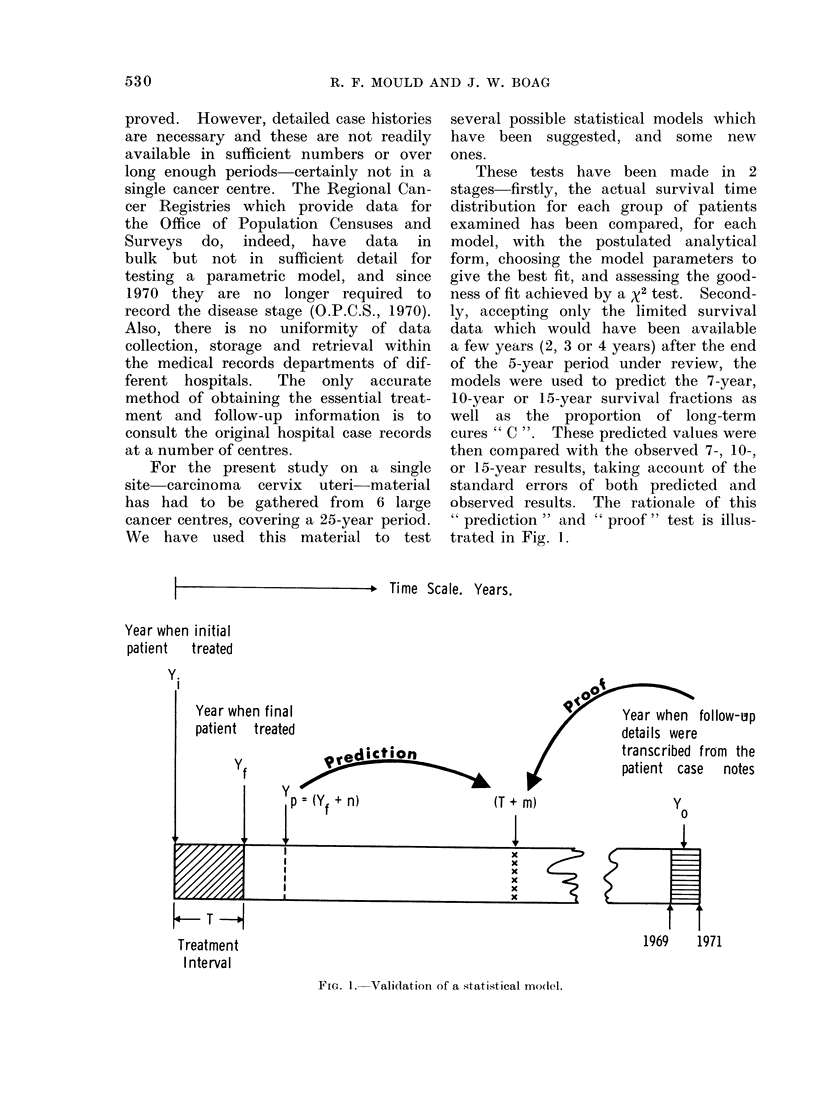

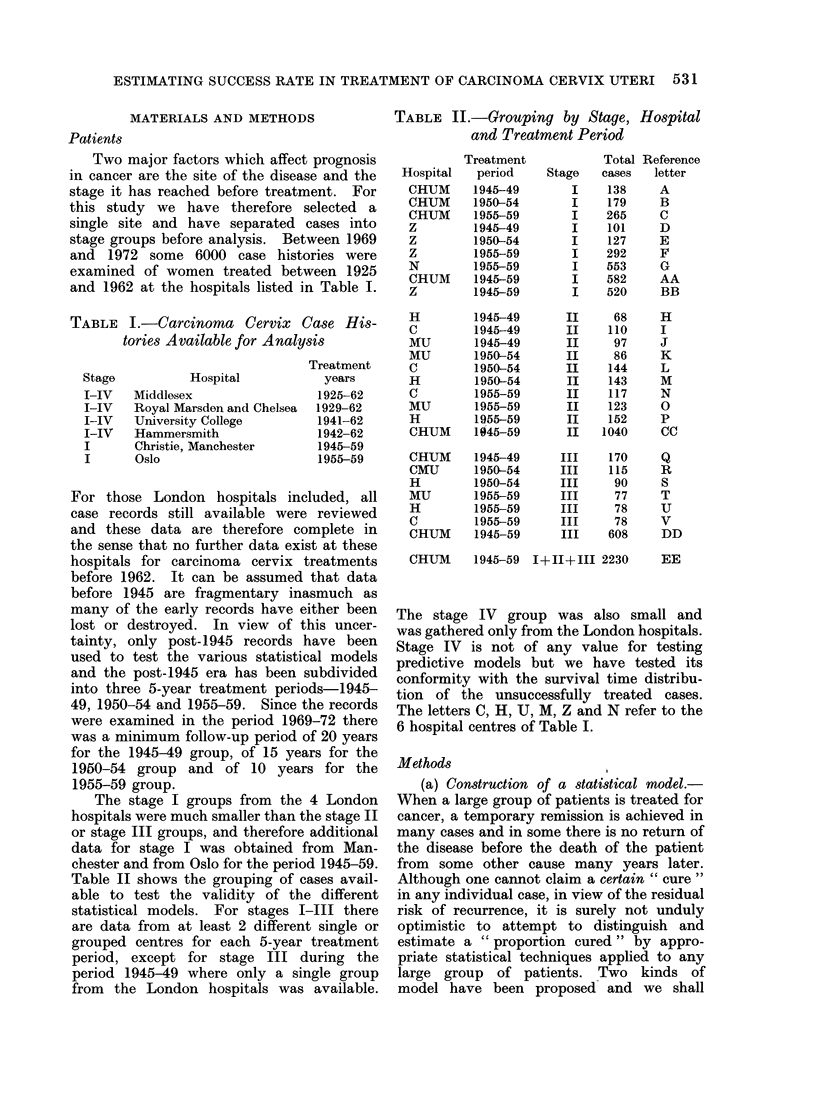

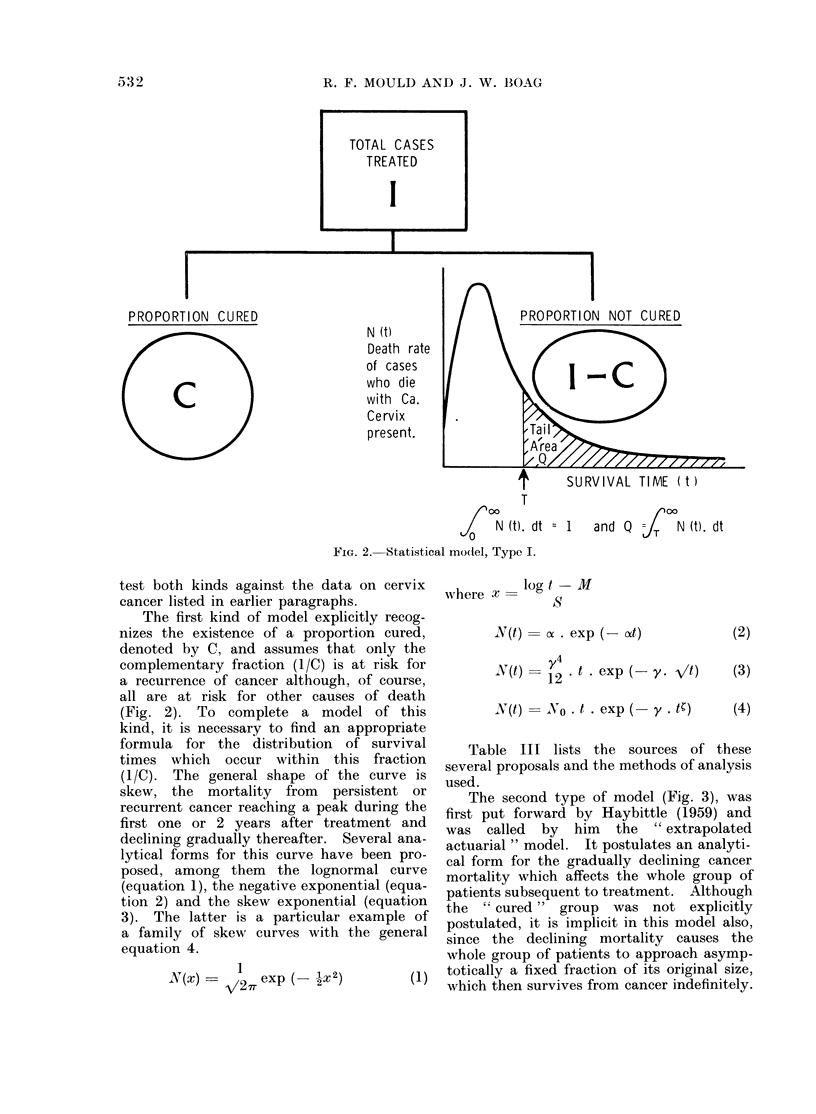

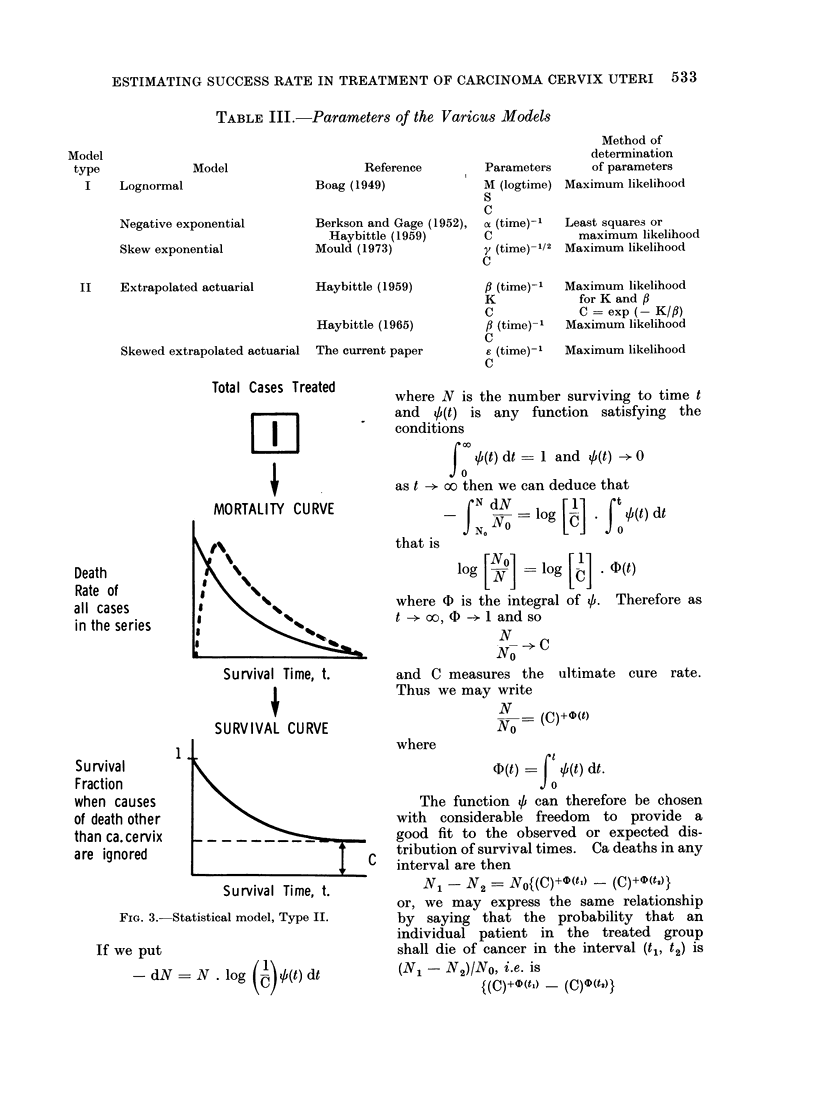

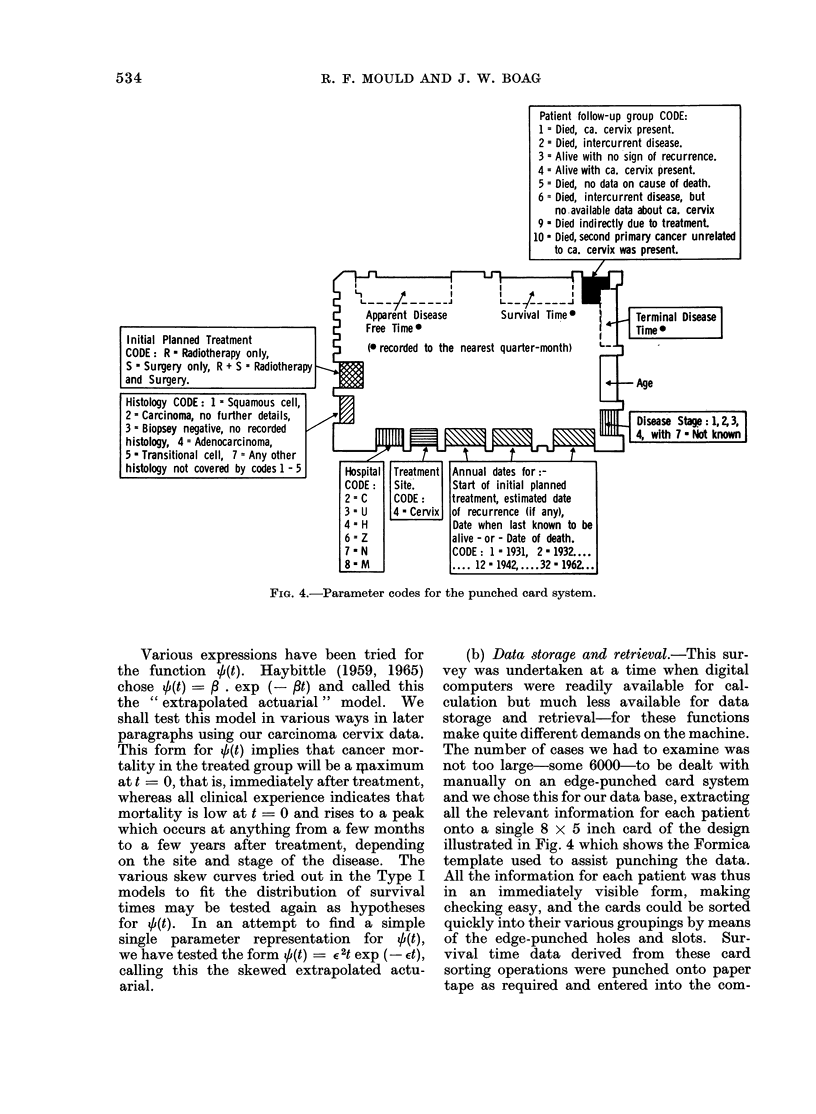

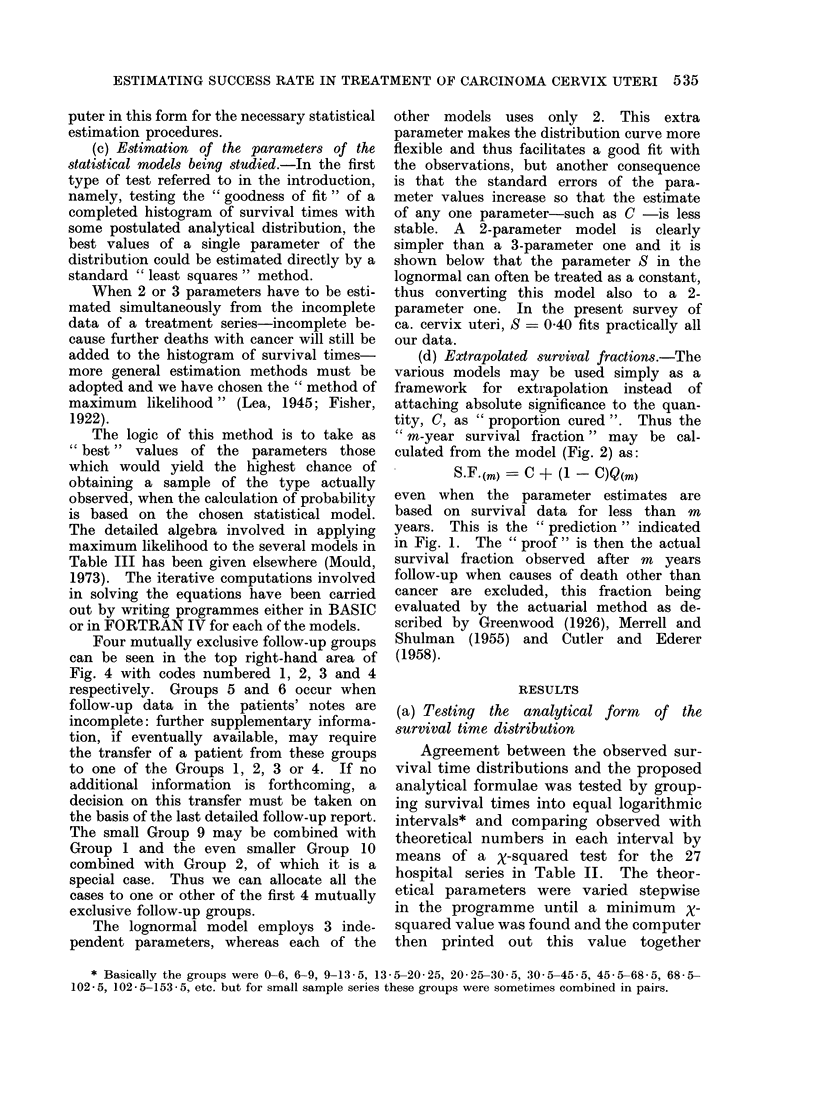

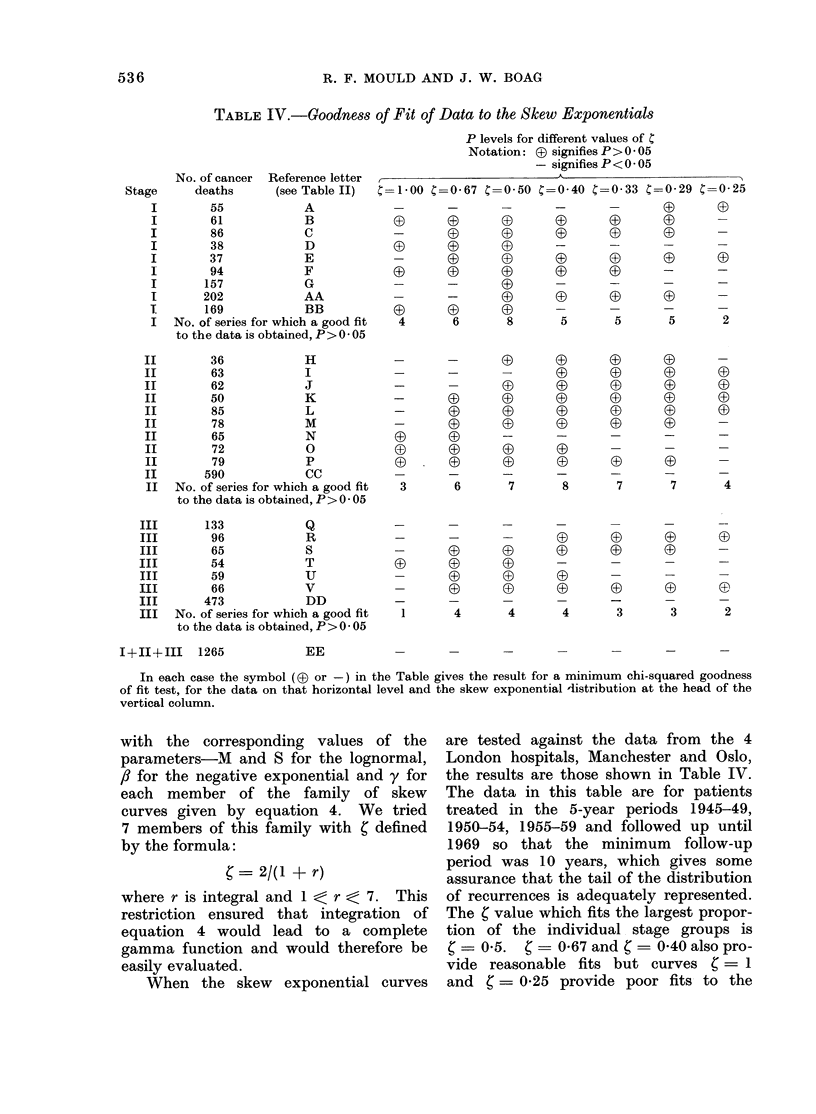

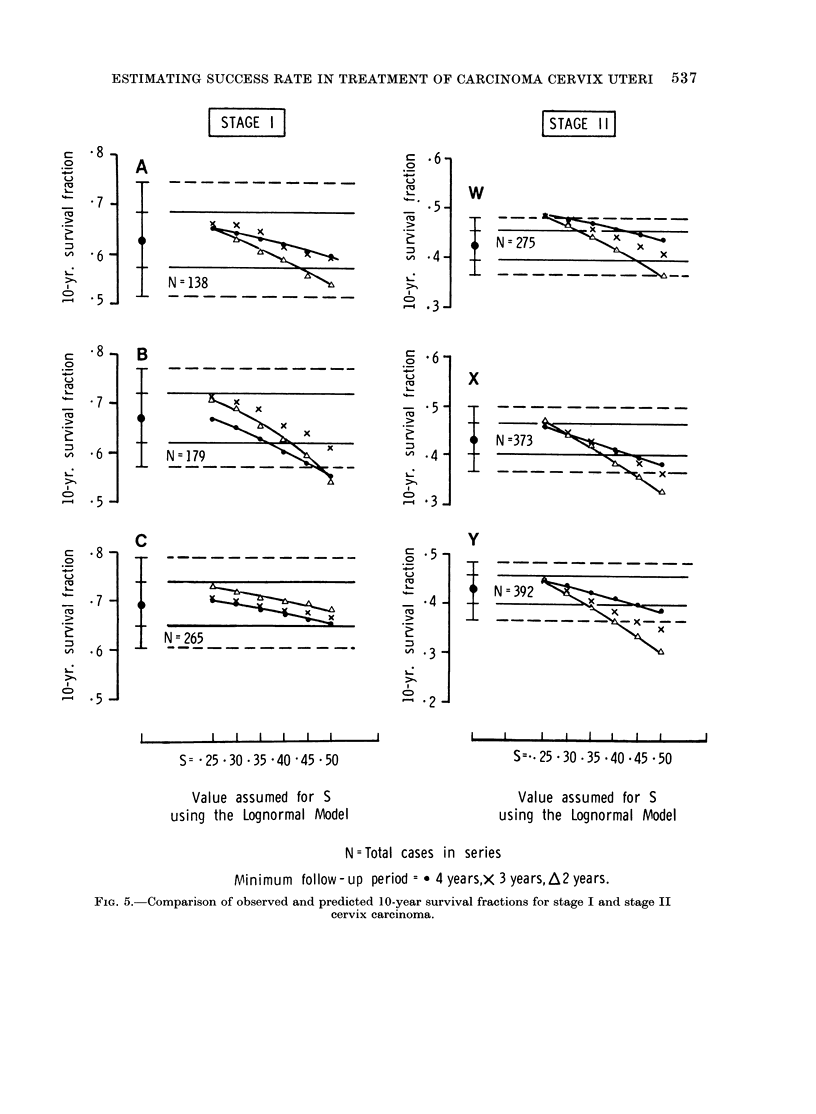

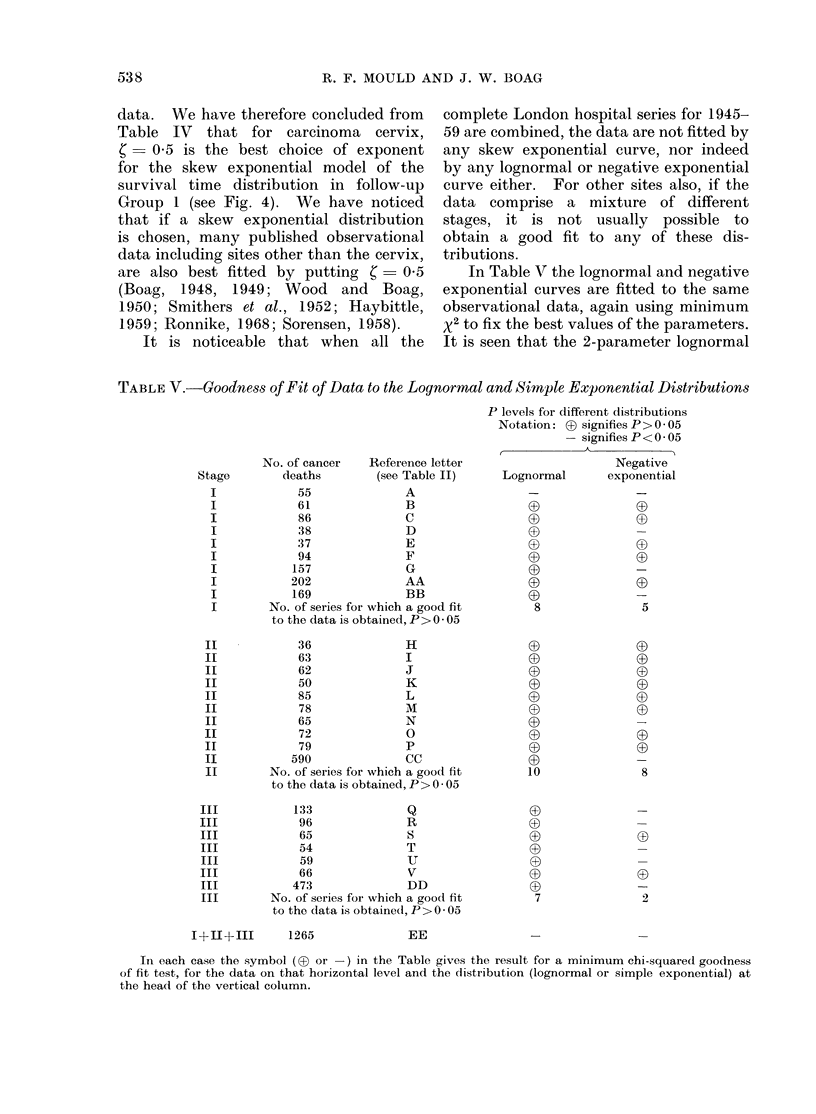

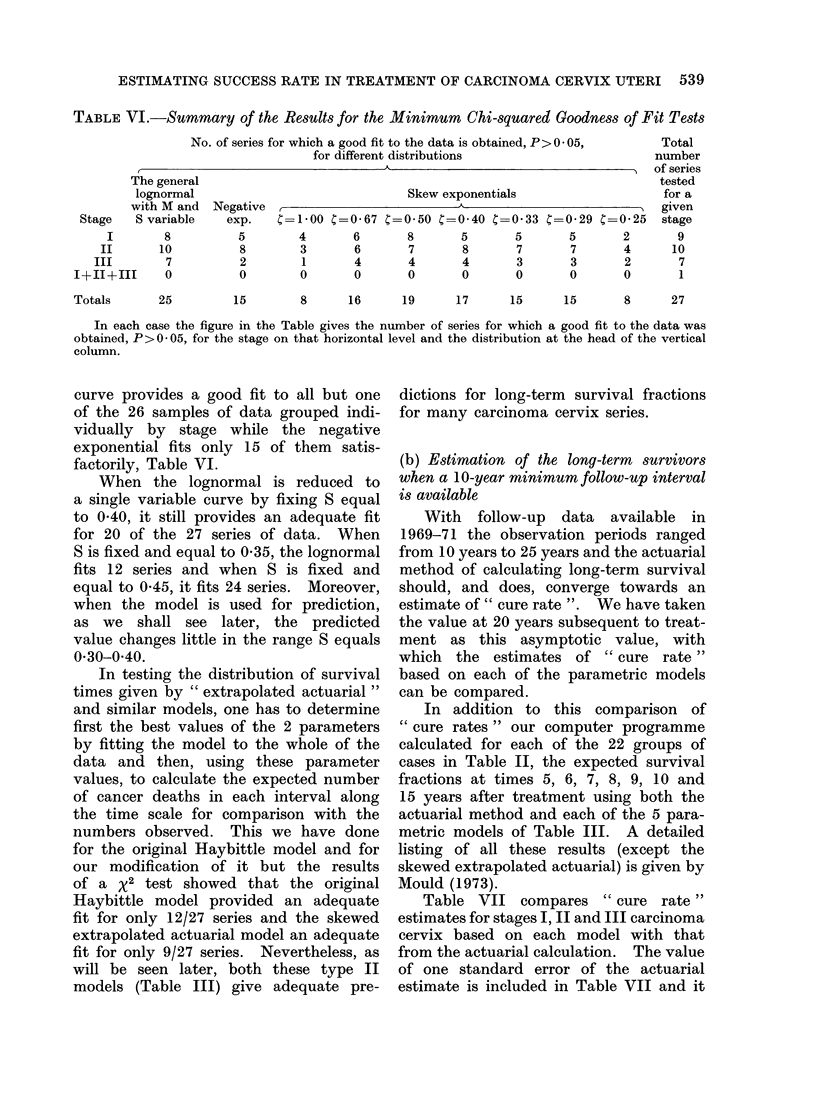

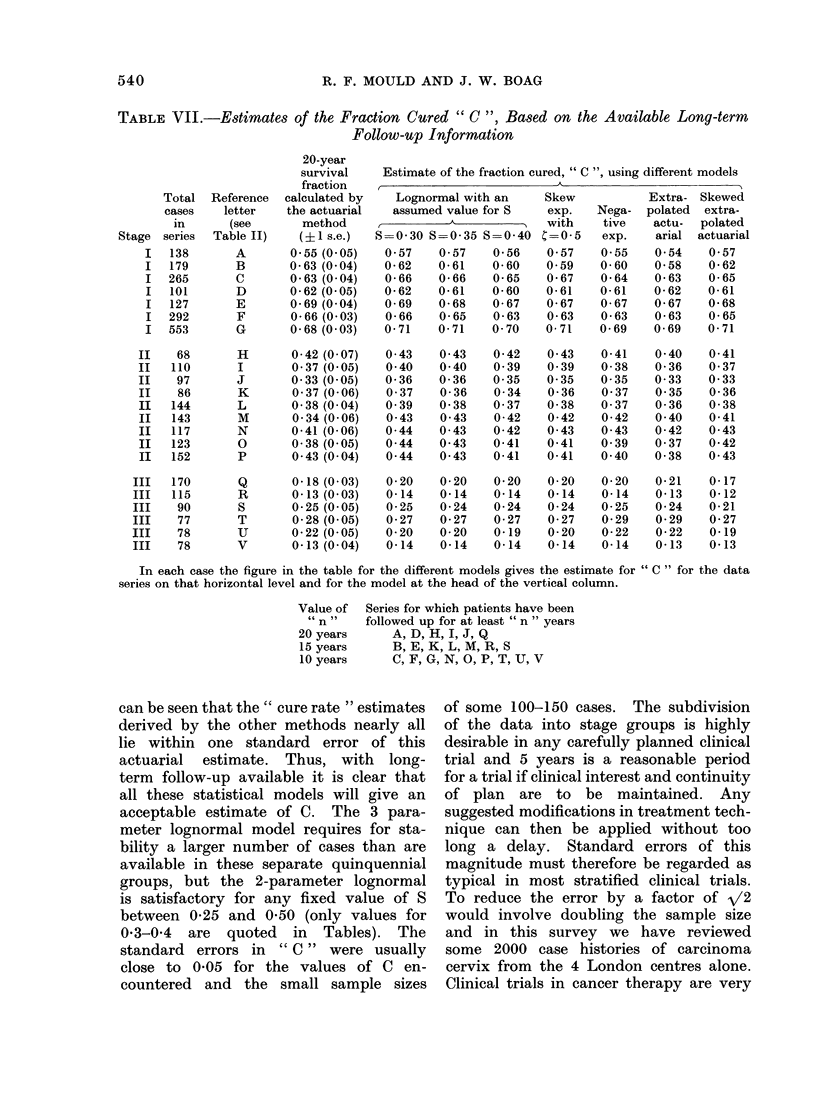

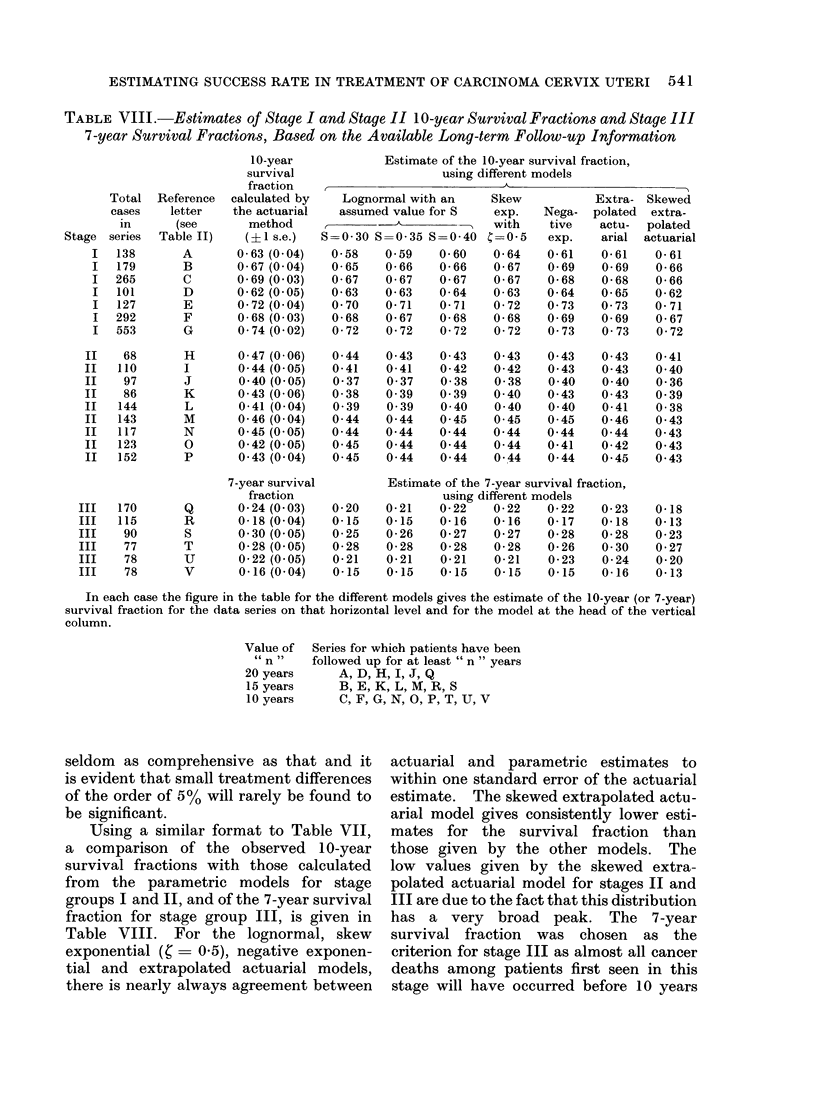

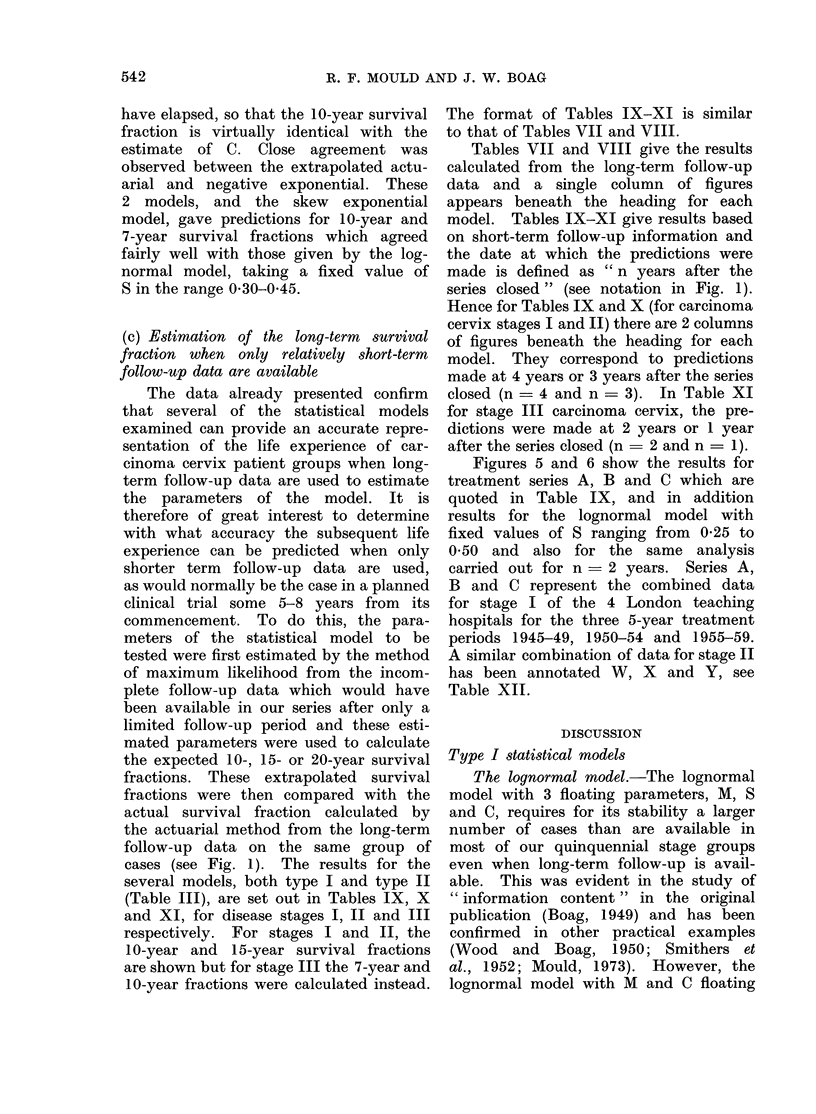

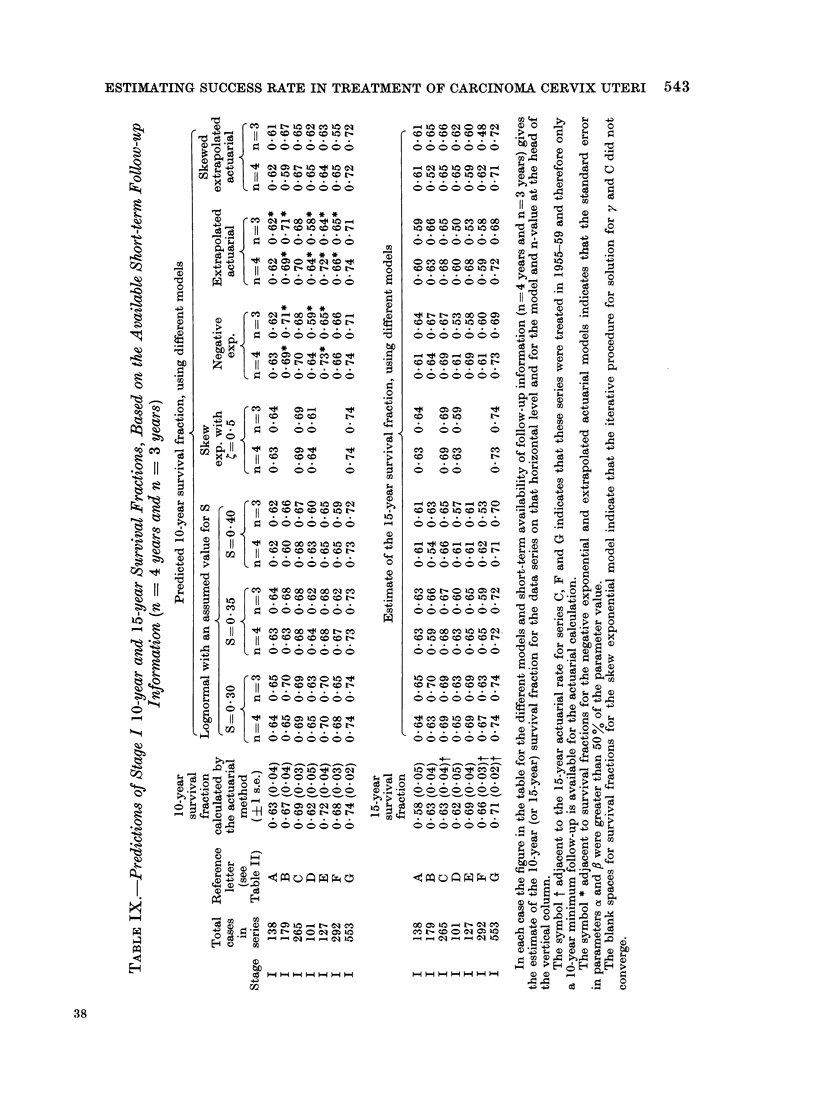

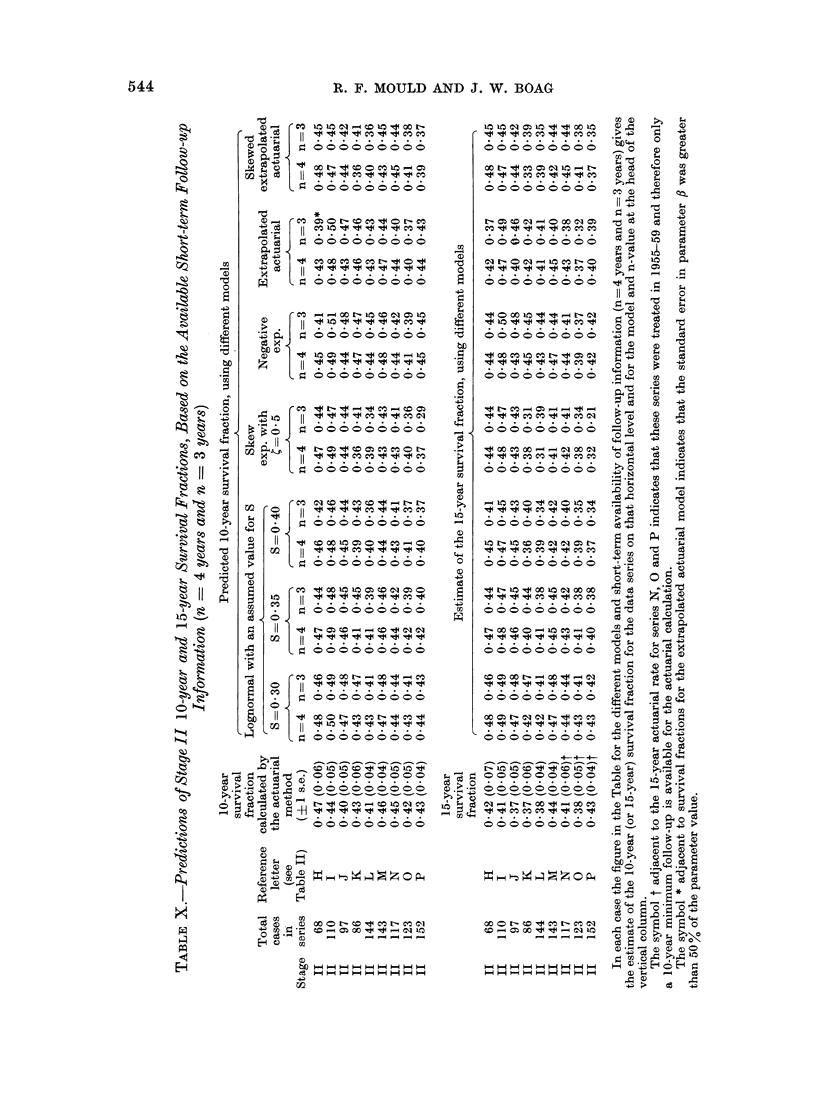

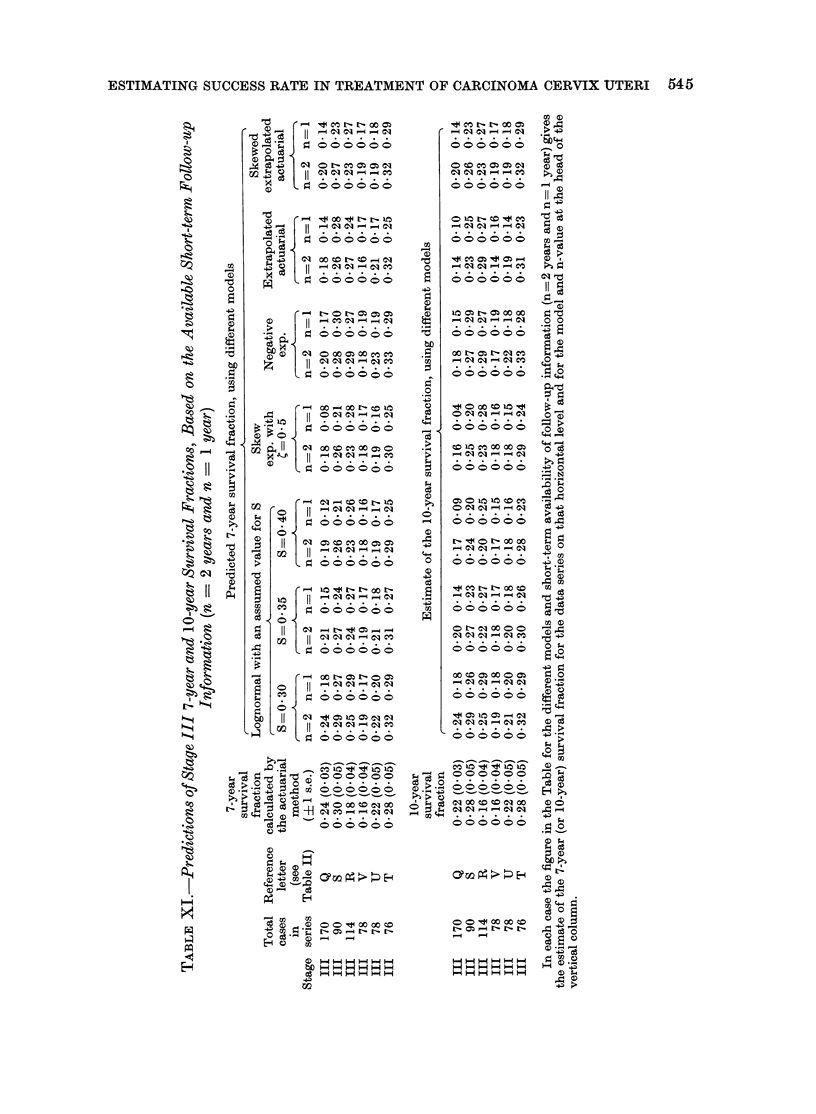

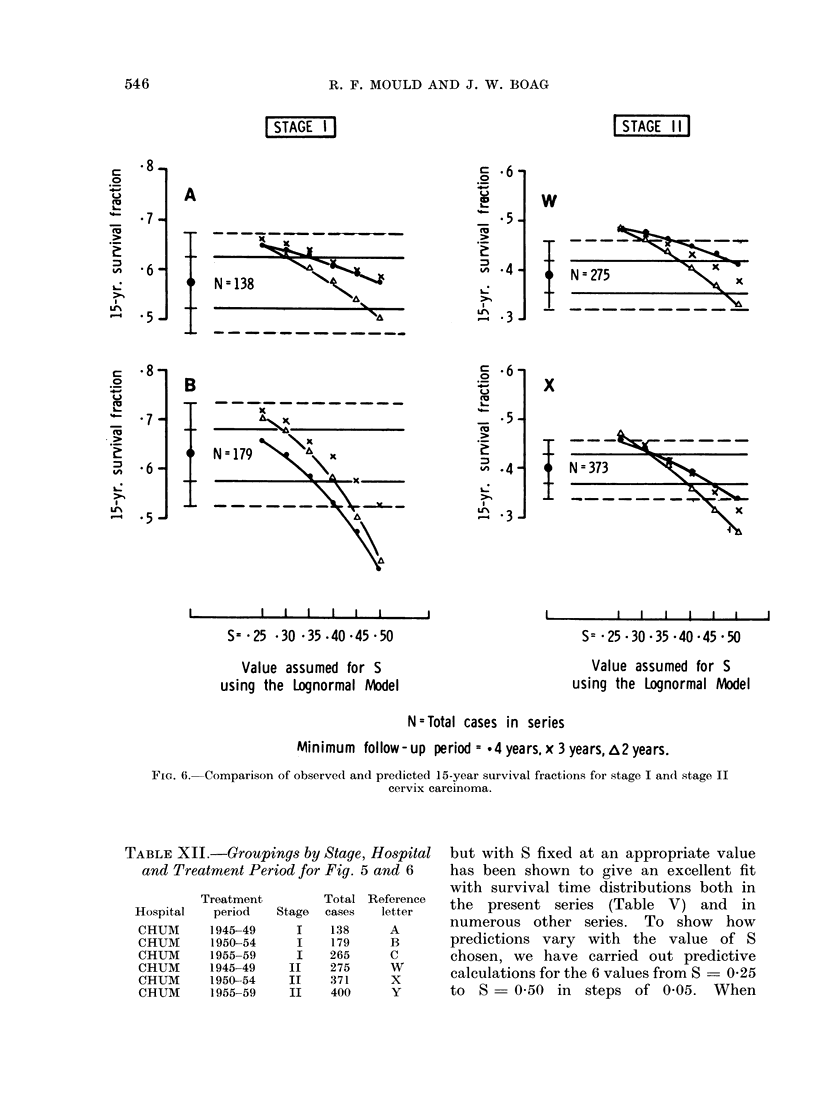

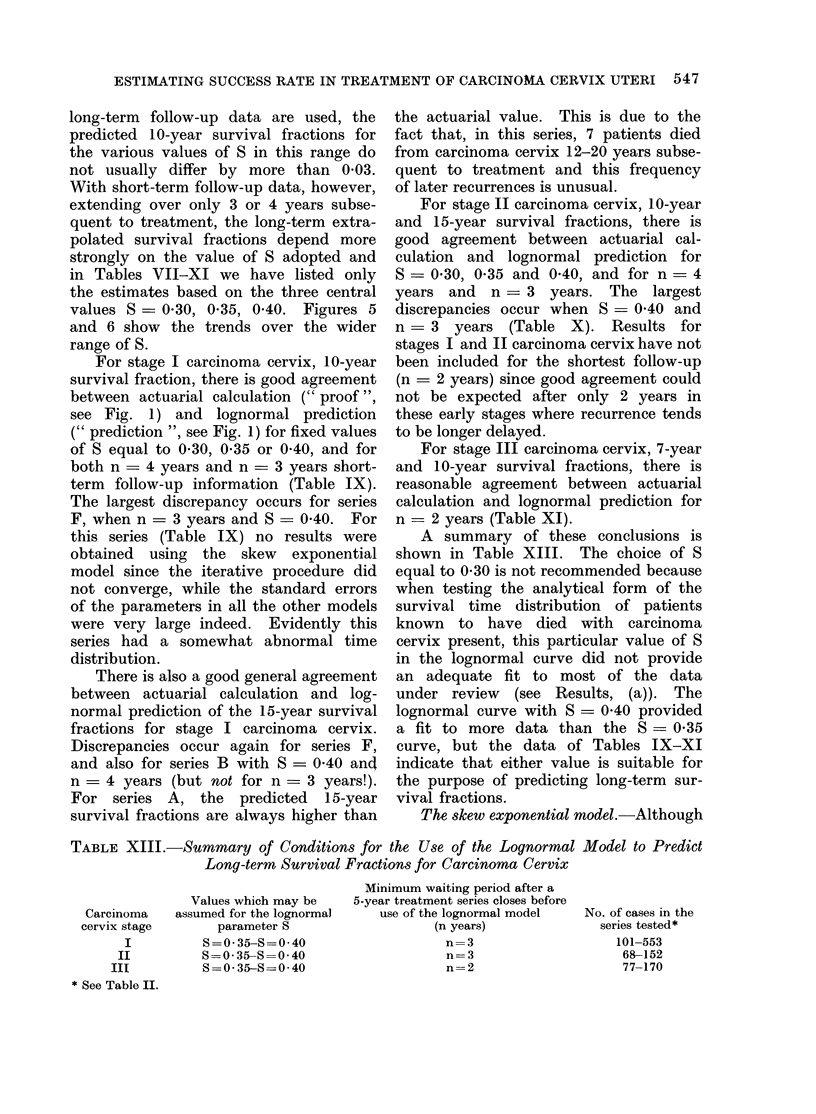

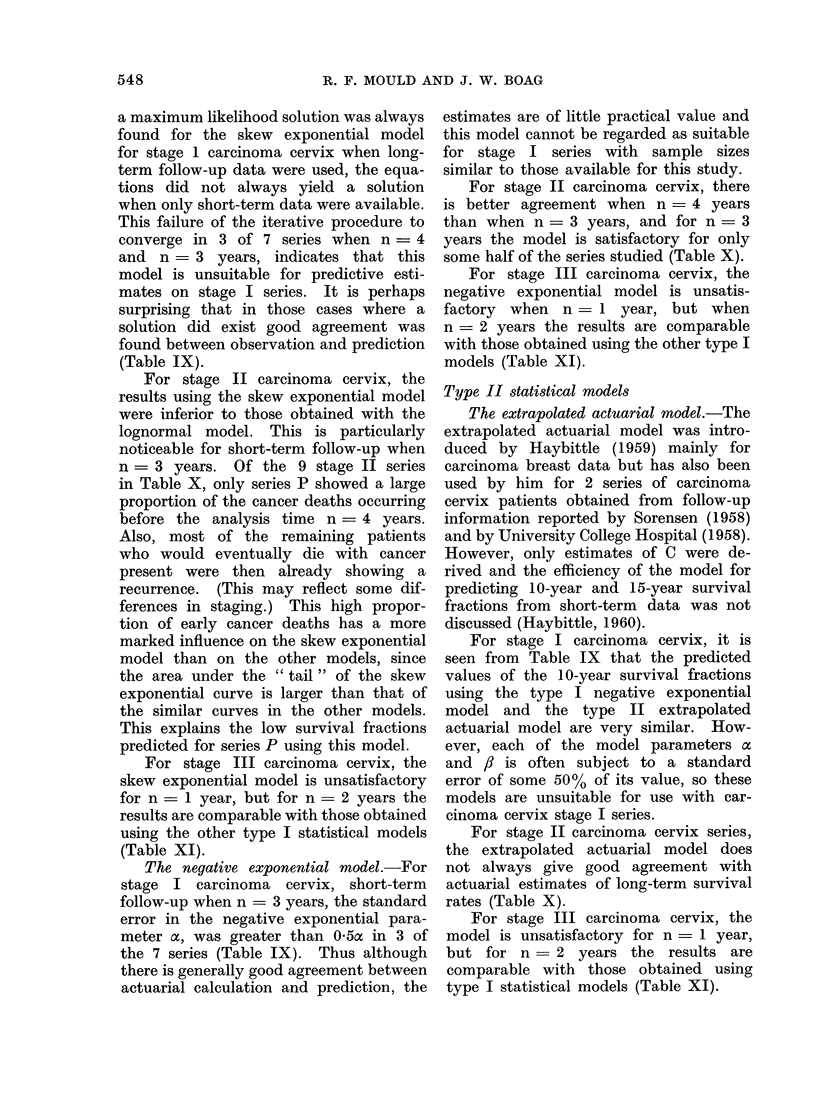

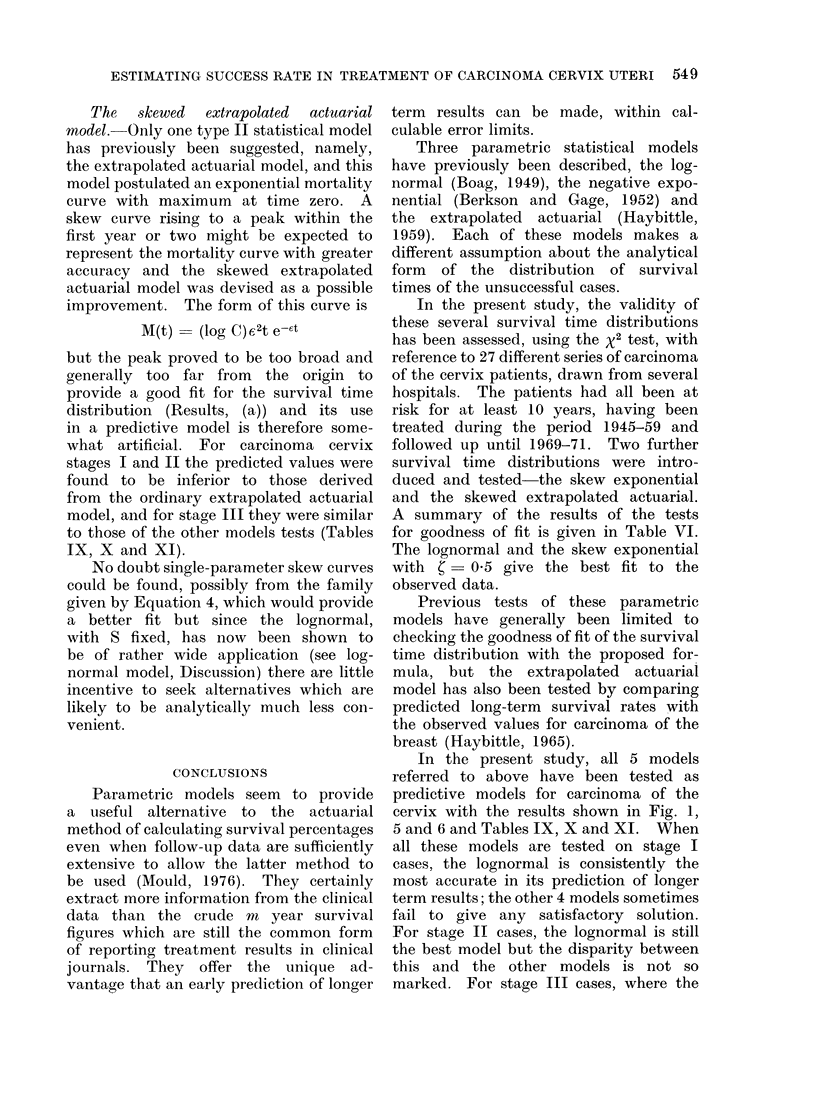

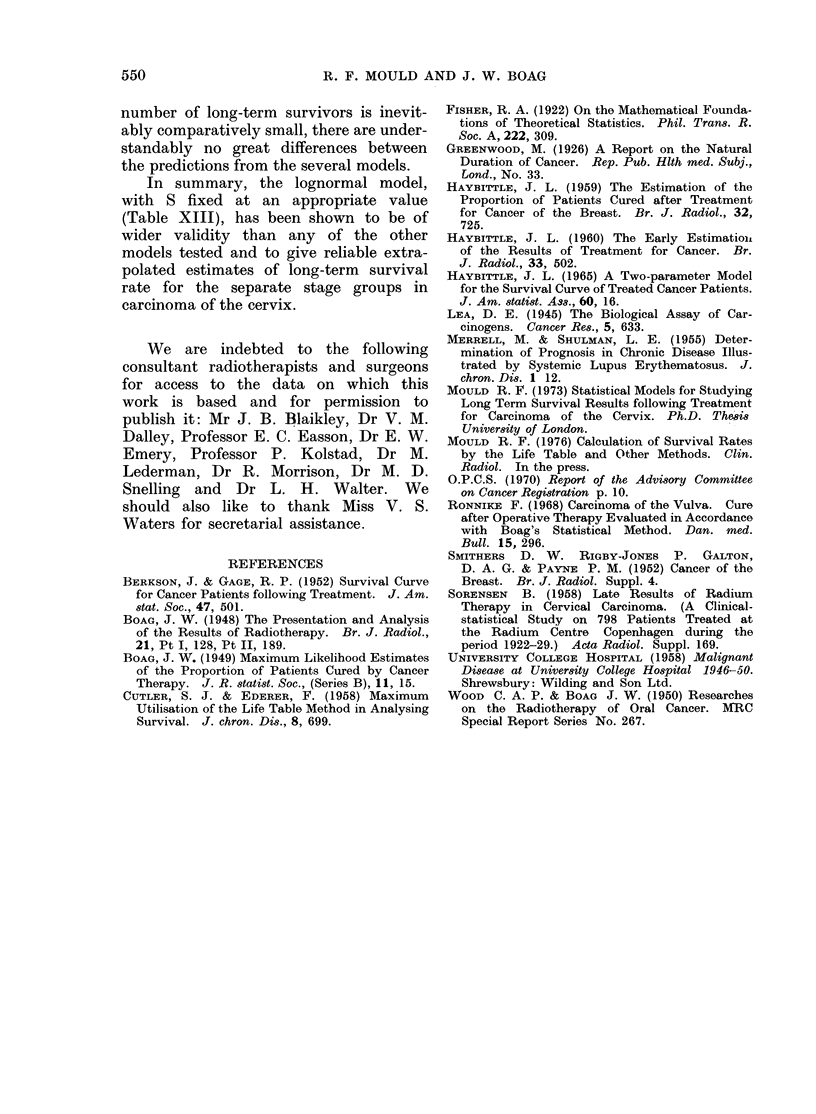

